# Genome-Wide Identification and Analysis of the Phosphoenolpyruvate Carboxylase Gene Family in *Suaeda aralocaspica*, an Annual Halophyte With Single-Cellular C_4_ Anatomy

**DOI:** 10.3389/fpls.2021.665279

**Published:** 2021-08-30

**Authors:** Jing Cao, Gang Cheng, Lu Wang, Tayier Maimaitijiang, Haiyan Lan

**Affiliations:** Xinjiang Key Laboratory of Biological Resources and Genetic Engineering, College of Life Science and Technology, Xinjiang University, Urumqi, China

**Keywords:** enzyme kinetics, genome-wide identification, PEPC, single-cellular C_4_ anatomy, *Suaeda aralocaspica*, transcriptional expression

## Abstract

Phosphoenolpyruvate carboxylase (PEPC) plays pivotal roles in the carbon fixation of photosynthesis and a variety of metabolic and stress pathways. *Suaeda aralocaspica* belongs to a single-cellular C_4_ species and carries out a photosynthetic pathway in an unusually elongated chlorenchyma cell, which is expected to have PEPCs with different characteristics. To identify the different isoforms of *PEPC* genes in *S. aralocaspica* and comparatively analyze their expression and regulation patterns as well as the biochemical and enzymatic properties in this study, we characterized a bacterial-type PEPC (BTPC; SaPEPC-4) in addition to the two plant-type PEPCs (PTPCs; SaPEPC-1 and SaPEPC-2) using a genome-wide identification. *SaPEPC-4* presented a lower expression level in all test combinations with an unknown function; two SaPTPCs showed distinct subcellular localizations and different spatiotemporal expression patterns but positively responded to abiotic stresses. Compared to *SaPEPC-2*, the expression of *SaPEPC-1* specifically in chlorenchyma cell tissues was much more active with the progression of development and under various stresses, particularly sensitive to light, implying the involvement of *SaPEPC-1* in a C_4_ photosynthetic pathway. In contrast, *SaPEPC-2* was more like a non-photosynthetic PEPC. The expression trends of two SaPTPCs in response to light, development, and abiotic stresses were also matched with the changes in PEPC activity *in vivo* (native) or *in vitro* (recombinant), and the biochemical properties of the two recombinant SaPTPCs were similar in response to various effectors while the catalytic efficiency, substrate affinity, and enzyme activity of SaPEPC-2 were higher than that of SaPEPC-1 *in vitro.* All the different properties between these two SaPTPCs might be involved in transcriptional (e.g., specific *cis*-elements), posttranscriptional [e.g., 5′-untranslated region (5′-UTR) secondary structure], or translational (e.g., PEPC phosphorylation/dephosphorylation) regulatory events. The comparative studies on the different isoforms of the PEPC gene family in *S. aralocaspica* may help to decipher their exact role in C_4_ photosynthesis, plant growth/development, and stress resistance.

## Introduction

Phosphoenolpyruvate carboxylase (PEPC, EC 4.1.1.31) is widely distributed in photosynthetic organisms such as vascular plants, algae, and photosynthetic bacteria. Plant PEPCs consist of a small gene family, which encodes several plant-type PEPCs (PTPCs) and at least one “distant relative” – bacterial-type PEPC (BTPC; Sánchez and Cejudo, 2003). All PTPCs encode approximately 100–110 kDa polypeptides with a conserved N-terminal seryl-phosphorylation domain and a distinguishing C-terminal tetrapeptide QNTG (Q-glutamine, N-asparagine, T-threonine, and G-glycine) signature (Izui et al., 2004; Xu et al., 2006). The 116–118 kDa-long polypeptides encoded by BTPCs show only about 40% identity with PTPC sequences, which harbor a prokaryotic-like (R/K) NTG (R-arginine, K-lysine) C-terminal tetrapeptide, but is lacking the N-terminal phosphorylatable Ser residue (Gennidakis et al., 2007; O’Leary et al., 2011a,b). With the completion of plant genome project and gene sequencing, numerous *PEPC* genes of different plant species have been identified, e.g., four *PEPCs* in *Arabidopsis* (Sánchez and Cejudo, 2003), five in tomato (Waseem and Ahmad, 2019), 10 in soybean (Wang et al., 2016), 5–9 in different peanut species (Yu et al., 2010; Pan et al., 2017; Tu et al., 2018), 6–11 among different cotton cultivars (Zhao et al., 2019), etc., however, limited information is available concerning *PEPC* from C_4_ species without Kranz anatomy. So far, except the cases of our previously uploaded two *PEPC* complementary DNA (cDNA) sequences of *Suaeda aralocaspica* in the GenBank database (KP985714.1 and KX009562.1 for *SaPEPC-1* and *SaPEPC-2*, respectively), only partial cDNA sequences of some *PEPC* genes were isolated from three single-cell (SC) C_4_ species (*S. aralocaspica*, *Bienertia cycloptera*, and *Bienertia sinuspersici*) (Lara et al., 2006; Rosnow et al., 2014). Currently, the introduction of the draft genome assembly of *S. aralocaspica* makes it possible to identify genome-wide *PEPC* genes in *S. aralocaspica* (Wang et al., 2019).

*Suaeda aralocaspica* (Bunge) Freitag and Schütze (Chenopodiaceae) is an annual halophyte, which is distributed in the southern margin of Junggar Basin in China, and is restricted to the saline–alkaline sandy soils of Gobi desert in central Asia (Commissione Redactorum Florae Xinjiangensis, 1994). It is the first terrestrial plant species discovered possessing the SC C_4_ photosynthetic pathway, since then, three more SC C_4_ species have been found in the genus *Bienertia* (Voznesenskaya et al., 2001; Sharpe and Offermann, 2014). In *S. aralocaspica*, the unusually long chlorenchyma cells are arranged in a single layer in the leaf, and the dimorphic chloroplasts have a spatially polar distribution between distal and proximal ends of the chlorenchyma cells, which is analogous to the Kranz anatomy but lacks the intervening cell wall (Edwards and Voznesenskaya, 2011). The key photosynthetic enzymes are biochemically compartmentalized in different regions of the cytoplasm (Voznesenskaya et al., 2004; for a review see Sharpe and Offermann, 2014), and it is speculated that the PEPCs might be different in types, enzymatic properties, and/or functions to those in Kranz C_4_ species. PEPC carboxylation rate is apparently higher in *S. aralocaspica* than in Kranz C_4_ species (Edwards et al., 2004; Smith et al., 2009; Liu et al., 2020); whereas some biochemical characteristics of PEPC in *S. aralocaspica* leaves are similar to Kranz C_4_ plants (i.e., protein phosphorylation in response to light/dark and some aspects of enzymatic kinetics) (Lara et al., 2006). In our previous study, a full-length (FL) cDNA sequence of *PEPC* gene in *S. aralocaspica* was isolated and termed as *SaPEPC-1* (GenBank: KP985714.1) according to the classification of Rosnow et al. (2014), and suggested its roles in development and stress tolerance in *S. aralocaspica* (Cheng et al., 2016). Recently, we cloned another *PEPC* gene cDNA sequence from *S. aralocaspica* termed as *SaPEPC-2* (GenBank: KX009562.1), which share 76.2% similarity with *SaPEPC-1*; we also achieved the complete genomic DNA sequences of *SaPEPC-1* and *SaPEPC-2* (GenBank: KU870624 and KU870625). So far, these two PEPCs remained uncertain in terms of category (C_3_ or C_4_ type), regulatory characteristics, biochemical properties, and the enzymatic kinetics. To dissect these questions, the major aims of the present study are: (1) To characterize all members of *PEPC* genes in *S. aralocaspica* at the genome-wide level. (2) To comparatively analyze the functional differences among *SaPEPC* isoforms in phylogenetic relations, gene structure, protein motifs, subcellular localization, transcriptional regulation, and enzyme biochemistry. (3) To investigate the contribution of different types of SaPEPCs in response to the development, light/dark, and abiotic stresses, as well as their role in a C_4_ photosynthetic pathway. The abovementioned goals needed to be achieved might help in further understanding of the roles of PEPC isoforms in C_4_ species.

## Materials and Methods

### Plant Materials and Cultivation

The mature seeds of *S. aralocaspica* were harvested from dry inflorescence of natural plants growing in the Gurbantunggut desert at Wujiaqu 103 regiment (44°37′N, 87°26′E; 423 mH) in October 2014, in the Xinjiang Uygur Autonomous Region, China. Seeds were air-dried indoor and cleaned and then stored at 4°C in a sealed brown paper bag. The brown seeds can germinate within 3 h upon contact with water, whereas the black seeds germinate much slower (Wang et al., 2008; He et al., 2013). However, the descendants from dimorphic seeds present no significant difference in morphological and physiological characteristics as well as gene expression patterns (Cao et al., 2015). Therefore, in this study, brown seeds were used in all the experiments.

#### Seed Germination and Treatments

To collect samples for total RNA extraction in seed germination, approximately 150 brown seeds were sown on the two layers of a filter paper in a 15 cm Petri dish, to which 20 ml of distilled water or other aqueous solutions were added. For different germination times, germinated seeds (seedlings) were harvested at 8 h, 12 h, 24 h, 2 days, 5 days, 10 days, and 15 days, respectively, and dry seeds at 0 h were used as control; for different tissues, cotyledons, hypocotyls, and radicles were sampled from the seedlings germinated for 7 days; and for different stress treatments, a filter paper was saturated with 20 ml of different concentrations (conc.) of aqueous solutions: NaCl (100, 300, and 500 mmol⋅L^–1^), isoosmotic mannitol (200, 600, and 1,000 mmol⋅L^–1^), and ABA (1, 5, and 10 μmol⋅L^–1^), respectively, only distilled water was used as control, and the seedlings were harvested after 7-day germination. All the above experiments were treated with normal light intensity (500 μmol⋅m^–2^⋅s^–1^) and under darkness (the Petri dish was properly wrapped with a foil to avoid light penetration). For different light treatments, all Petri dishes were subjected to a photoperiod of 16 h light/8 h dark with the light intensities of 30, 300, and 900 μmol⋅m^–2^⋅s^–1^, respectively, and the seedlings were harvested after 7-day germination.

#### Seedling Growth and Treatments

The brown seeds were sown in pots containing perlite: vermiculite (1:3, v/v) in a growth chamber, under the conditions of a 16 h light/8 h dark photoperiod with the light intensity of 500–700 μmol⋅m^–2^⋅s^–1^, a temperature regime of 24–30°C, and a relative humidity of 10–20%. The pot soil was carefully sprayed with distilled water or other aqueous solutions by a mini-sprinkler till the distilled water (solutions) was drained out from the bottom of the pot when the draining solution volume and the pot volume were approximately the same, a fresh distilled water (corresponding solutions) was transferred into the pot in a tray for 2 h to keep the soil saturated with distilled water (solutions) during initiation, consequently supplemented with the distilled water (corresponding solutions) at an interval of 1 week. Seedlings cultivated with distilled water were used as control [in addition to the half-strength Hoagland solution (Arnon and Hoagland, 1940) at an interval of 2–3 weeks]. For different tissues, the leaves, stems, and roots on day 15 (seedling) and day 90 (adult plant) after emergence were collected; for different developmental stages, whole seedlings (for gene expression analysis) or cotyledons/leaves (for PEPC activity measurement) were harvested on day 3, 15, 30, and 60, respectively, after emergence; for salt stress, seedlings were treated with the half-strength Hoagland solution containing 100, 300, or 500 mmol⋅L^–1^ NaCl and the cotyledons/leaves were harvested on day 15 after emergence; for drought stress, seedlings at 30 days of emergence were subjected to natural drought for 7, 14, and 28 days, respectively; and for different photoperiods, 60-day-old plants after emergence were cultivated in a greenhouse during the continual sunny days, on the 2nd or 3rd day, the leaves on the top of the plants were harvested at an interval of 2 h from the morning at 8:00 to the evening at 22:00 within the same day. Supplementary Table 1 provides the detailed descriptions for different experimental designs and sampling times.

All samples were immediately frozen in liquid nitrogen on harvesting and then stored at −80°C until use. Four biological replicates were applied to each treatment.

### Identification of *PEPC* Genes in *S. aralocaspica*

To identify the potential members of *PEPC* gene family in *S. aralocaspica* genome, firstly, the amino acid sequences of the four PEPCs in *Arabidopsis thaliana* were used as a query to conduct a local BLASTP search by a cut-off *E*-value of 1 × 10^–5^ (Supplementary Table 2). Subsequently, the Hidden Markov Model- (HMM-) based profile of the PEPCase domain (PF00311) obtained from the Pfam database^1^ was used to verify the candidates of *PEPC* gene homologs by HMMER^2^ and SMART^3^ searches. Finally, the candidates of *PEPC* homologs were further validated in the presence of a PEPC family domain (IPR021135), a lysine active site (IPR018129), and a histidine active site (IPR033129) on the InterProScan website^4^.

### Multi-Sequence Alignment and Phylogenetic Analysis

Multiple alignments were performed by FL amino acid sequences using the ClustalW program of MEGA X with the default settings (Kumar et al., 2018). The phylogenetic tree of PEPC proteins from 27 plant species was constructed using the unrooted neighbor-joining method of MEGA X with the following parameters: Poisson correction, pairwise deletion, and a bootstrap analysis with 1,000 replicates. The amino acid sequences of the other 26 representative species (Monocots: *Brachypodium distachyon*, *Oryza sativa*, *Panicum virgatum*, *Setaria italica*, *Sorghum bicolor*, and *Zea mays*; Dicots: *A. thaliana*, *Arachis hypogaea*, *Brassica rapa*, *Chenopodium quinoa*, *Glycine max*, *Gossypium raimondii*, *Linum usitatissimum*, *Manihot esculenta*, *Medicago truncatula*, *Phaseolus vulgaris*, *Populus trichocarpa*, *Ricinus communis*, and *Solanum lycopersicum*; Pteridophytes: *Selaginella moellendorffii*; Bryophyte: *Physcomitrella patens*; Photosynthetic algae: *Chlamydomonas reinhardtii*, *Coccomyxa subellipsoidea*, *Micromonas pusilla*, *Ostreococcus lucimarinus*, and *Volvox carteri*) were acquired from the Phytozome database^5^. Supplementary Table 2 provides a detailed description of the abovementioned proteins and their corresponding accession numbers.

### Analyses of Gene Structures, Conserved Motifs, and *Cis*-Regulatory Elements

For gene structure analysis, the exons and introns of *PEPC* genes were identified due to the alignment of cDNA sequences with the corresponding genomic DNA sequences and were illustrated using the GSDS 2.0 server^6^. The MEME program^7^ was employed to identify and analyze the conserved motifs of PEPC proteins with default parameters, and the maximum number of motifs to be detected was set as 10. The *cis*-regulatory elements in the promoter sequences (2,500 bp upstream of the start codon) of *PEPC* genes were identified using the PlantCARE database^8^. The Mfold RNA/DNA folding program^9^ was used to predict the secondary structure of a 5′-untranslated region (5′-UTR) of *PEPC* genes. The MEME and PlantCARE results were visualized using the TBtools software (Chen et al., 2020). Meanwhile, the theoretical molecular weight (*MW*), isoelectric point (*pI*), and grand average of hydropathicity (GRAVY) of PEPC candidates were predicted using the ExPASy website^10^.

### Determination of Subcellular Localization

Plant-mPLoc^11^ and YLoc+^12^ websites were used to predict the subcellular localization of candidate PEPCs in *S. aralocaspica*, which were further verified by a transient expression system in tobacco epidermal cells. The open reading frame (ORF) sequence of *SaPEPC-1* or *SaPEPC-2* (with the stop codon deletion) was fused to an enhanced green fluorescent protein (eGFP) ORF, and then inserted into the plant binary expression vector pCAMBIA1300, which resulted in the construct *35S::SaPEPCs-eGFP*. The primers used for vector construction are presented in Supplementary Table 3.

The abovementioned recombinant vectors were transformed into *Agrobacterium tumefaciens* strain GV3101 through a CaCl_2_ method. The correct single colony was inoculated in a YEB medium (50 mg⋅L^–1^ kanamycin, 50 mg⋅L^–1^ gentamicin, and 50 mg⋅L^–1^ rifampicin) and cultivated with a shaking speed of 220 rpm at 28°C till the OD_595_ value reached the range of 0.8–1.0. Then, 2 ml of cultures were removed for centrifugation at 12,000 rpm for 2 min to collect cells, which were then resuspended in an infiltration buffer (10 mmol⋅L^–1^ MES, 10 mmol⋅L^–1^ MgCl_2_, and 150 μmol⋅L^–1^ acetosyringone) at a final concentration of OD_595_ = 0.8. *A. tumefaciens* suspension (A) of the abovementioned constructs was evenly mixed with *35S::CBL-RFP*/GV3101 (B) [Calcineurin B-like protein 1 (CBL1), located on the plasma membrane, used as control] (Batistic et al., 2010) and *35S::P19*/GV3101 (C) (P19 protein: promoted protein expression) suspensions with a volume ratio of 450 μl (A): 300 μl (B): 300 μl (C); for *SaPEPC-2*, *35S::ABI5-BFP*/GV3101 (D) [abscisic acid insensitive 5 (ABI5), located in the nucleus, used as control] (Bensmihen et al., 2005) was also included in a volume ratio of 450 μl (A): 300 μl (B): 300 μl (C): 300 μl (D) of *A. tumefaciens* suspension. The mixture was held at room temperature for 2–3 h in the dark before use. About 5- to 6-week-old *Nicotiana benthamiana* plants were prepared for infiltration. The tip end of a syringe (without a needle) is placed against the underside of the leaf (in avoidance of the veins) with one finger supporting on the upper side, then gently pressing the syringe to infiltrate *A. tumefaciens* mixture into the fresh leaf and labeled the infiltration area for further recognition. The treated plants were held in the dark overnight, and were then transferred to the normal growth conditions for another 48 h. The fluorescent signals in the leaf of *N. benthamiana* were examined and photographed using the Zeiss LSM 800 confocal microscope (Carl Zeiss, Jena, Germany).

### Assay of the Promoter Activity

A series of 5′-deletions of *SaPEPC-1* and *SaPEPC-2* promoters were generated according to the predicted sites of light-response elements (Supplementary Figure 1). FL of each promoter was truncated into six fragments and labeled as fragments 1–6 in an order from the smaller to the larger upstream of ATG. Seven specific upstream primers (Ppc-FLF and Ppc-F1 to F6) and a single downstream primer (Ppc-FLR) were designed for each fragment in a *SaPEPC* promoter. In addition, another downstream primer Ppc-R_TSS_ was designed to combine with the upstream primer Ppc-F6 to amplify the 5′-flank regions from the transcription start site (TSS), which was labeled as TSS (Supplementary Table 3). Following the digestion with endonucleases *Hin*dIII and *Bam*HI, the CaMV35S promoter sequence was replaced by the abovementioned fragments in the plant expression vector pBI121 to drive β-glucuronidase (*GUS*) gene. The recombinant constructs were transformed into *A. tumefaciens* strain EHA105 for the transient expression test in *N. benthamiana*, the manipulation was similar to that in the determination of subcellular localization except that the suspension of each construct was not necessary to mix with *35S::CBL1*/GV3101, *35S::ABI5*/GV3101, and *35S::P19*/GV3101 strains. Treated tobacco plants were exposed to normal illumination (500 μmol⋅m^–2^⋅s^–1^) for 3 days in a growth chamber, and the darkness group was placed in the dark cabinet to avoid light until sampling. A GUS fluorometric assay was performed according to Jefferson et al. (1987). All leaves were ground in liquid nitrogen and homogenized in 1.0 ml of the freshly prepared GUS extraction buffer [200 mmol⋅L^–1^ NaH_2_PO_4_, 200 mmol⋅L^–1^ Na_2_HPO_4_, 500 mmol⋅L^–1^ ethylenediaminetetraacetic acid (EDTA), 0.1% (v/v) Triton X-100, 0.1% (v/v) β-mercaptoethanol, and 10% (w/v) sodium dodecyl sulfate (SDS)]. After centrifugation at 12,000 rpm, 4°C for 15 min, the supernatant was employed to determine the GUS activity using 4-methylumbelliferyl glucuronide (4-MUG) as a substrate. The fluorescence of 4-methylumbelliferone (4-MU) produced by GUS-catalyzed hydrolysis was measured by the FLx800^TM^ Fluorescence Reader (BioTek, Winooski, VT, United States). The protein concentration of the supernatant was assessed by the method of Bradford (1976) using bovine serum albumin (BSA) as the standard. GUS activity was normalized to the protein concentration of each supernatant extract and calculated as pmol of 4-MU per microgram of soluble protein per minute.

### Analysis of Gene Expression Profile

Gene expression profile was analyzed based on public released data. Published gene expression data sets in different tissues (matured leaves, stems, roots, and fruits) of *S. aralocaspica* were downloaded from the NCBI (SRA: SRP128359; BioProject: JNA428881) (Wang et al., 2019). The RNA sequencing (RNA-Seq) data sets of dimorphic seeds in the germination of *S. aralocaspica* were obtained from the BioProject of PRJNA325861 (Wang et al., 2017). Gene expression levels were estimated by the fragments per kilobase of exon per million mapped reads (FPKM) values using the Cufflinks software (Trapnell et al., 2012). The heatmap was generated using the TBtools software (Chen et al., 2020), the color scale represents FPKM counts, and the ratios were expressed as log2 transformed.

### Quantitative Real-Time PCR

To validate the transcriptomic data, we performed a quantitative real-time PCR (qRT-PCR) to analyze the expression of *SaPEPC-1* and *SaPEPC-2* genes. Total RNA was extracted from the collected plant samples using the E.Z.N.A.^®^ Plant RNA Kit (Cat. R6827, OMEGA, Norcross, GA, United States) according to the instructions of the manufacturer. RNA conc. and absorbance ratios (A_260_/A_280_ and A_260_/A_230_) were measured using the NanoDrop^®^ ND-1000 spectrophotometer (Thermo Fisher Scientific, Waltham, MA, United States). Each reverse transcription reaction was performed with 1 μg of total RNA in a final volume of 20 μl using the M-MLV RTase cDNA Synthesis Kit (D6130, TaKaRa, Shiga, Japan) with a 2.5 μmol⋅L^–1^ oligo (dT) primer following the instructions of the manufacturer. cDNA was stored at −20°C until use. qRT-PCR was carried out using GoTaqR^®^ qPCR Master Mix (Promega, Madison, WI, United States) in the GeneAmp^®^ 7500 Real-Time PCR System (ABI, Vernon, CA, United States). Gene-specific primers of *SaPEPC-1* and *SaPEPC-2* were designed using the Primer-Blast tools^13^ (Supplementary Table 3). To ensure the amplification of the desired product, a melt-curve analysis was performed to determine that only a single peak was present to represent a unique PCR product as per the MIQE guidelines (Bustin et al., 2009). Standard curves were generated for each primer to assess efficiency, and all primers had a value of efficiency between 1.9 and 2.1 (Supplementary Data). β*-tubulin* gene of *S. aralocaspica* was used as an internal reference (Cao et al., 2016). The reaction mixture consisted of 1 μl cDNA samples, 0.5 μl each of the forward and reverse primers (10 μmol⋅L^–1^), 10 μl GoTaqR^®^ qPCR master mix, and 8 μl nuclease-free H_2_O in a final volume of 20 μl. qRT-PCR was performed as follows: 2 min initial denaturation at 95°C, followed by 40 cycles at 95°C for 15 s, and 60°C for 1 min. Four biological replicates with two technical replicates for each treatment were applied, and the data were analyzed using the 2^–ΔΔ*Cq*^ method (Taylor et al., 2019). The final value of relative quantification was described as a normalized fold change in the gene expression of each target gene compared to the control. Data were expressed as geometric mean ± 95% CI of four biological replicates for each treatment.

### Expression and Detection of Recombinant Protein

The ORF of *SaPEPC-1* or *SaPEPC-2* was inserted into the prokaryotic expression vector pET28a. The primers used for vector construction are shown in Supplementary Table 3. The recombinant plasmids pET28a-*SaPEPCs* were transformed into *Escherichia coli* Transetta (DE3) strain. The positive clones were sequenced and cultivated in a liquid LB medium supplemented with 100 mg⋅L^–1^ kanamycin and 0.8 mmol⋅L^–1^ isopropyl β-D-1-thiogalactopyranoside (IPTG) at 37°C for 4 h to induce the expression of *SaPEPCs*. The cell pellets of the recombinant strains were ultrasonically treated, and the total amount of proteins was harvested by centrifuging at 12,000 *g*, 4°C for 10 min. The precipitation was resuspended and resolved by –SDS-polyacrylamide gel electrophoresis (SDS-PAGE), and the recombinant protein was detected by an immunoblot according to the following steps: upon separation on 10% (w/v) PAGE, the proteins were electroblotted onto a polyvinylidene fluoride (PVDF) membrane, which was then blocked overnight at 4°C in a Tris-buffered saline (TBS) buffer (20 mmol⋅L^–1^ Tris–HCl, pH 7.5; 150 mmol⋅L^–1^ NaCl) containing 5% (w/v) powdered milk. After the incubation with mouse anti-His monoclonal antibody (1:1000 diluted) for 2 h at 37°C, the membrane was washed four times in a TBS buffer, and then incubated with a 1:1000 diluted goat anti-mouse IgG secondary antibody. The 3,3-diaminobenzidine (DAB) was added as a chromogen for staining.

### Assay of Stress Tolerance of Recombinant Protein

The recombinant (Transetta: pET-28a-*SaPEPCs*) and control (Transetta: pET-28a) strains were inoculated in a fresh LB medium containing 100 mg⋅L^–1^ kanamycin and cultured overnight at 37°C, which (1% of the culture) was then reinoculated to a fresh LB medium (in addition of 100 mg⋅L^–1^ kanamycin) and cultivated for about 4 h till the OD_600_ value reached 0.5. After the addition of 0.8 mmol⋅L^–1^ IPTG, the cultures were incubated for another 4 h at 37°C. About 1% of the diluted culture (0.8 OD_600_) was inoculated into a 50 ml fresh LB medium (in addition of 100 mg⋅L^–1^ kanamycin) with the supplement of 400 mmol⋅L^–1^ NaCl, 10% (w/v) PEG 6000, or 25 μmol⋅L^–1^ methyl viologen (MV; to mimic the oxidative stress). For the test of acid or base response, the pH value of a LB medium was adjusted to 5.0. Except for the case of temperature assay (at 30°C), all other cultures were incubated at 37°C with a shaking speed of 220 rpm overnight. For the measurement of the time course of growth under different abiotic stresses, cultures (10 ml) were harvested at an interval of 3 h to a total of 12 h to measure the enzyme activity.

### Assay of the Kinetic Property and Stability of Enzymes

The recombinant proteins SaPEPC-1 and SaPEPC-2 were purified under native conditions with an Ni-NTA agarose resin (Qiagen, Hilden, Germany) according to the protocol of the manufacturer. After the determination of the concentration of the purified proteins by using PEP as a substrate, the enzyme activity was determined by the addition of the different conc. of PEP (0.5, 1, 3, 4, and 5 mmol⋅L^–1^), NaHCO_3_ (0.5, 3, 5, 10, and 20 mmol⋅L^–1^), and MgCl_2_ (0.5, 3, 5, 10, and 20 mmol⋅L^–1^) at pH 8.0 and 25°C. The Michaelis constant *K*_m_ and the maximum reaction rate *V*_max_ were calculated according to the Lineweaver–Burk plot method, and the catalytic constant *K*_cat_ (*K*_cat_ = *V*_max/_enzyme concentration) was calculated to measure the speed of an enzymatic reaction. The heat stability of the purified SaPEPCs was determined by measuring the enzyme activity at various temperatures (15–55°C). The pH stability was determined by an incubation with a 50 mmol⋅L^–1^ Tris–HCl reaction buffer under the pH values from 7.0 to 10.0 at 25°C. The metal ion stability was determined using a reaction buffer containing 10 mmol⋅L^–1^ EDTA and 10 mmol⋅L^–1^ metal ions (Cu^2+^, Al^3+^, and Mn^2+^), respectively. The deionized water was used as the control, and the enzyme activity was measured at pH 8.0 and 25°C. The effect of metabolic effectors on enzyme activity was estimated in the presence of varying amounts of allosteric activators (0, 5, 10, 20, and 40 mmol⋅L^–1^ glucose-6-phosphate or glycine) and inhibitors (0, 2, 5, 10, 15, 20, and 40 mmol⋅L^–1^ L-malate) at pH 8.0 and 25°C.

### Measurement of PEPC Enzyme Activity

For PEPC activity in *S. aralocaspica*, leaves (approximately 0.1 g) were homogenized on ice with 1.0 ml of an extraction buffer containing 100 mmol⋅L^–1^ Tris-H_2_SO_4_ (pH 8.2), 7 mmol⋅L^–1^ β-mercaptoethanol, 1 mmol⋅L^–1^ EDTA, and 5% (v/v) glycerol. The homogenate was then centrifuged at 2,000 rpm, 4°C for 20 min. The supernatant was immediately used for the assay of PEPC activity according to the protocols described by Cao et al. (2015). For the enzymatic activity of recombinant proteins, the 10 ml samples from the different bacterial cultures were centrifuged at 12,000 *g* for 10 min, cell pellets were washed with a phosphate buffer, then sonicated for 10 times of 3 s of each with an interval of 10 s and centrifuged at 12,000 *g*, 4°C for 10 min. The supernatant was employed as a crude enzyme and kept on ice for immediate use. Enzyme activity was measured as described by Cheng et al. (2016). The absorbance of reaction mixtures was recorded by monitoring NADH oxidation at 340 nm on an UV-3010 spectrophotometer (Shimadzu, Kyoto, Japan). The total amount of proteins was determined at 595 nm (Bradford, 1976). One unit of PEPC enzyme activity was defined as an optical density value decrease of 0.01 per minute (Nomenclature Committee of the International Union Of Biochemistry (NC-IUB)., 1979).

### Immunoblot Analysis of Photosynthetic Enzymes

Leaves (∼0.2 g) of *S. aralocaspica* were used for the extraction of soluble proteins according to the method described by Koteyeva et al. (2011). The supernatant was mixed with a loading buffer [250 mmol⋅L^–1^ Tris–HCl, pH 6.8, 10% (w/v) SDS, 50% (v/v) glycerol, 5% (v/v) β-mercaptoethanol, and 0.5% (w/v) bromphenol blue] as 4:1 in volume and boiled for 10 min after centrifugation at 10,000 *g*, 4°C for 10 min, the supernatant was subjected to the SDS-PAGE analysis. Protein concentration was determined using the Bradford Protein Assay Kit (Cat. PC0010, Solarbio, Beijing, China). The resolved protein samples (10 mg of each) were transferred to a PVDF membrane for an immunoblot analysis of the photosynthetic enzymes. All the primary antibodies used in this study were raised against the predicted optimal epitopic antigens of the conserved amino acid sequences of PEPC, pyruvate orthophosphate dikinase (PPDK), and ribulose-1,5-bisphosphate carboxylase/oxygenase (Rubisco) from *S. aralocaspica*, the amino acid residues of the epitopic antigens, and the working dilution of these antibodies were as follows: anti-SaPEPC-C (EKLSSIDAQLR, common to PEPC) IgG (1:500), anti-SaPEPC-M {[EKLS(pS)IDAQLR], for the detection of the phosphorylation of the serine residue in this sequence of PEPC} IgG (1:500), anti-SaPPDK (KLATEKGRAAKPSL) IgG (1:200), and anti-SaRubisco large subunit (RBCL) (QARNEGRDLAREGN) IgG (1:500). The secondary antibody goat anti-rabbit IgG (conjugated horseradish peroxidase) (1:2000) was used for detection. Bound antibodies were visualized by enhanced chemiluminescence (Biosharp, Beijing, China), and the images were acquired by an luminescent image analyzer (FUJIFILM LAS-4000, Tokyo, Japan).

### Statistical Analysis

All data were plotted using GraphPad Prism Version 7.0 (GraphPad Software, San Diego, CA, United States) and analyzed using SPSS version 26.0 (SPSS Inc., Chicago, IL, United States). Univariate scatterplots displaying parametric data were present as mean and SD (Weissgerber et al., 2015). One-way ANOVA was used to test the significance of different treatments, and Tukey’s HSD test was performed for multiple comparisons to determine significant differences between the samples at 0.05, 0.01, 0.001, and 0.0001 significance levels. When the homogeneity of variance assumption was not met, differences were analyzed using Welch’s ANOVA and Games Howell *post hoc* test. Statistically significant differences between the groups at 0.05 significance level were determined by an unpaired Student’s *t*-test (homoscedastic) or unpaired Student’s *t*-test using Welch’s correction (heteroscedastic) of a two-tailed distribution (McDonald, 2014).

## Results

### Identification of *PEPC* Gene Family in *S. aralocaspica*

A total of three putative *PEPC* genes were identified by a local BLASTP search of *S. aralocaspica* genome, the deduced proteins were subjected to Pfam, SMART, and InterProScan databases to analyze the domains and active sites. Three non-redundant genes (GOSA_00009595-RA, GOSA_00006741-RA, and GOSA_00018957-RA) were confirmed as *SaPEPCs*, the first two were recognized as *SaPEPC-1* (GenBank: KP985714.1) and *SaPEPC-2* (GenBank: KX009562.1), respectively, which were identified in our previous work; the third one was similar to *AtPPC4* in *Arabidopsis* and denominated as *SaPEPC-4*. Detailed information of *SaPEPC* family members is shown in Table 1.

**TABLE 1:** Characteristics of *PEPC* gene family in *S. aralocaspica*.

Gene name	Gene model name	Gene length (bp)	ORF (bp)	Exons Introns	Protein					Subcellular location	
					Size (aa)	MW (kDa)	*pI*	GRAVY	Aliphatic index	Plant-mPLoc	YLoc+
*SaPEPC*-1	GOSA_ 00009595-RA	5651	2901	10/9	966	110.2	6.10	−0.396	90.06	Cytoplasm	Cytoplasm (60%) or nucleus (33.7%)
*SaPEPC*-2	GOSA_ 00006741-RA	6701	2901	10/9	966	110.0	5.61	−0.390	88.35	Cytoplasm	Cytoplasm (74.1%) or nucleus (15.5%)
*SaPEPC-4*	GOSA_ 00018957-RA	10637	3099	20/19	1032	116.9	6.27	−0.463	88.25	Cytoplasm	Cytoplasm (74.6%) or nucleus (23.2%)

### Phylogenetic Analysis and Sequence Alignment of PEPCs in *S. aralocaspica*

**FIGURE 1:**
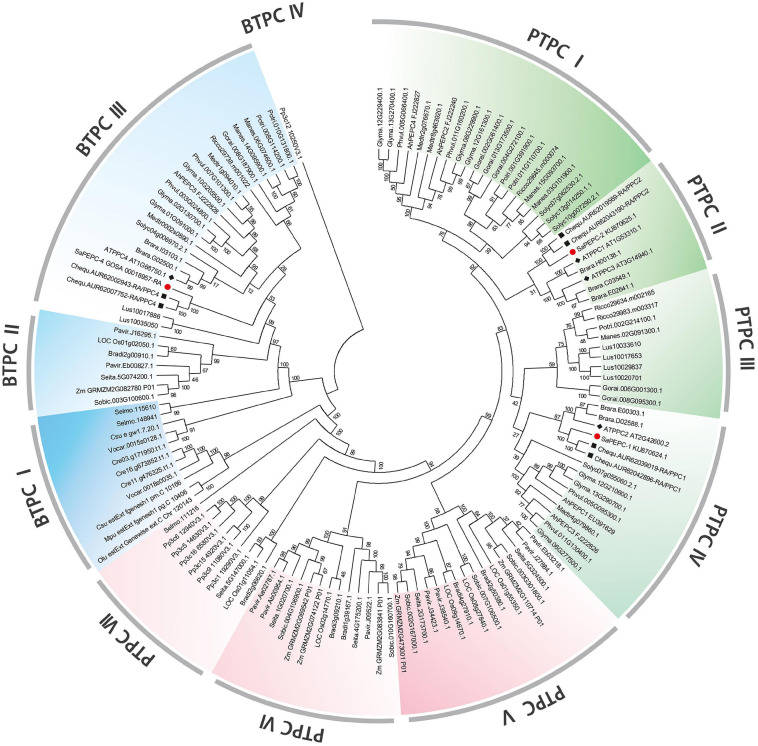
Phylogenetic tree of phosphoenolpyruvate carboxylase (*PEPC*) gene family in different plant species. Green (dicots) and red (monocots, mosses, and ferns) portions are the plant types of PEPC (PTPC); the blue part is a bacterial type of PEPC (BTPC). At, *Arabidopsis thaliana*; Ah, *Arachis hypogaea*; Bradi, *Brachypodium distachyon*; Brara, *Brassica rapa*; Chequ, *Chenopodium quinoa*; Cre, *Chlamydomonas reinhardtii*; Csu, *Coccomyxa subellipsoidea*; Glyma, *Glycine max*; Gorai, *Gossypium raimondii*; Lus, *Linum usitatissimum*; Manes, *Manihot esculenta*; Mt, *Medicago truncatula*; Mpu, *Micromonas pusilla*; LOC Os, *Oryza sativa*; Olu, *Ostreococcus lucimarinus*; Pavir, *Panicum virgatum*; Phvul, *Phaseolus vulgaris*; Pp3c, *Physcomitrella patens*; Potri, *Populus trichocarpa*; Rc, *Ricinus communis*; Selmo, *Selaginella moellendorffii*; Seita, *Setaria italica*; Solyc, *Solanum lycopersicum*; Sobic, *Sorghum bicolor*; Sa, *Suaeda aralocaspica*; Vocar, *Volvox carteri*; and Zm, *Zea mays*. The symbols of red circle, black box, and black diamond box indicate the *PEPC* gene family members in *S. aralocaspica*, *C. quinoa*, and *Arabidopsis*, respectively.

To investigate the evolutionary relationship between SaPEPCs and PEPCs of other 26 representative eukaryotic species, including dicots, monocots, ferns, mosses, and algae, we constructed an unrooted neighbor-joining phylogenetic tree using 135 PEPC proteins (Figure 1). The results indicate that all PEPC family members can be categorized into two distinct clades: PTPCs and BTPCs, about 70% of the PEPCs were PTPCs. Similar distribution patterns of PTPCs and BTPCs were also found in different species (Supplementary Table 4). In the PTPC subfamily, compared with a dicot branch, monocots, mosses, and ferns were gathered together and formed another independent branch, which could further be divided into seven groups (PTPC I–PTPC VII). BTPC subfamily was distinctly classified into four groups (BTPC I–BTPC IV). Phylogenetic analysis also identified some closely related orthologous PEPCs among *S. aralocaspica*, *A. thaliana*, and *C. quinoa* (a C_3_ plant, Geissler et al., 2015): SaPEPC-1, AtPPC2, and CqPPC1 were located on the same branch of PTPC IV; SaPEPC-2, AtPPC1/3, and CqPPC2 were assigned to PTPC II; whereas SaPEPC-4 was grouped into AtPPC4 and CqPPC4 cluster in BTPC III, suggesting that an ancestral set of PEPCs may exist prior to the divergence of *S. aralocaspica*, *A. thaliana*, and *C. quinoa*.

**FIGURE 2:**
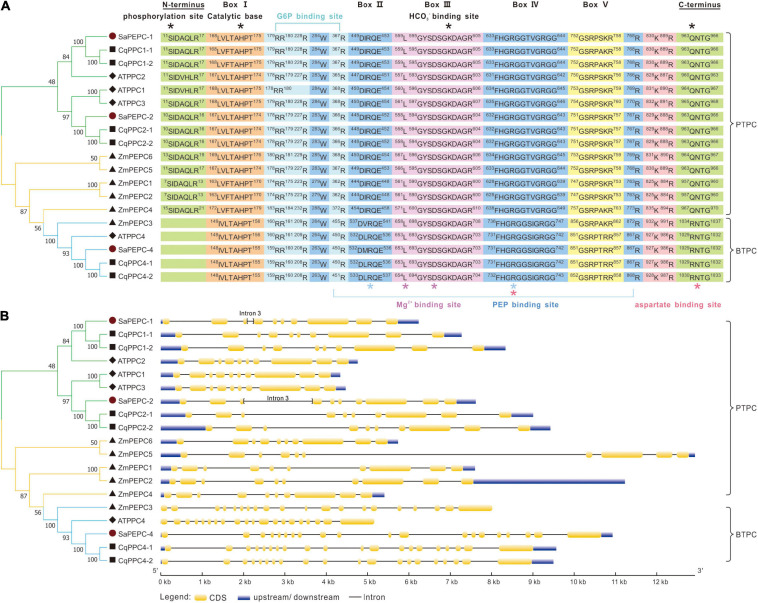
Sequence alignment **(A)** and gene structure analysis **(B)** of PEPCs among *S. aralocaspica*, *A. thaliana*, *C. quinoa*, and *Z. mays*. Box I–V are conserved sequences responsible for the activity of PEPC enzyme. *: Indicates the key domains of the catalytic reaction and binding sites for the substrate and inhibitor.

The amino acid sequence alignment among SaPEPCs, AtPPCs, CqPPCs, and ZmPEPCs showed that they shared typical conserved domains and functional sites in PTPC and BTPC genes. However, no N-terminal phosphorylation domain (SIDAQLR) was found in the polypeptide deduced from *SaPEPC-4*, *CqPPC4*, *AtPPC4*, and *ZmPEPC3* genes, instead of harboring an RNTG tetrapeptide at the C-terminus, which was commonly found in BTPC (Figure 2A).

### Gene Structure Analysis of *PEPCs*

Different *PEPC* genes in the same species displayed great discrepancies in size. *PEPC* genes in PTPC II/IV groups contained 10 exons, whereas it was 20 in BTPC III (Figure 2B). By predicting the exon–intron structure in 27 plant species (including *S. aralocaspica*), we found that the length of PTPC genes was from about 4 to 13 kb, and that of BTPC genes ranged from 7.5 to 14 kb; whereas the length of *PEPC* exons was similar in the same branch, and the number of exons/introns was conserved (Supplementary Figure 2). The PTPC genes of dicots, monocots, and ferns contained 10–12 exons and 9–11 introns, whereas the BTPC genes contained 18–21 exons and 17–20 introns. The moss PTPC genes generally consisted of 11–14 exons and 10–13 introns, except for one member with only five exons and four introns; while the independent branches of moss BTPCs contained 32 exons and 31 introns. All algal *PEPCs* belonged to BTPC, generally containing 20–30 exons and 19–29 introns, but exceptionally, only one exon was found in that of *Ostreococcus lucimarinus* and *Microcystis aeruginosa*.

Although the exon number was largely different, the exon length of PTPC genes was similar to that of BTPC, especially exons 8, 9, and 10; whereas the intron length differed greatly, indicating that the size of *PEPC* genes largely depends on the introns. Similarly, in *S. aralocaspica*, although the length of coding region of *SaPEPC-1* and *SaPEPC-2* (both were 2,901 bp) and the number of exons/introns (10/9) were conserved, the size of *SaPEPC-2* was 1,000 bp longer than that of *SaPEPC-1*, for the 3rd intron of the former was about 10 times longer than that of the latter (Figure 2B).

### Conserved Motif Analysis of PEPC Family

Multi-sequence alignment showed that PEPCs were highly conserved among *S. aralocaspica*, *A. thaliana*, and *C. quinoa*. The analysis of the top 10 conserved motifs of 135 PEPCs from 27 plant species revealed that all these motifs bore the prints (IPR021135) of PEPCase family, besides, motif-4 and motif-8 also contained the histidine (IPR033129) and lysine (IPR018129) active sites, respectively (Supplementary Figure 3 and Supplementary Table 5). SaPEPCs and other 121 PEPCs contained all these 10 motifs and were arranged in the same order, whereas the other five PTPCs and six BTPCs were lacking some of these motifs (Supplementary Table 6). It suggests that PEPCs in different species are generally conserved in gene structures, protein domains, and functional motifs but can also be genetically diverse.

### Subcellular Localization of SaPEPCs

**FIGURE 3:**
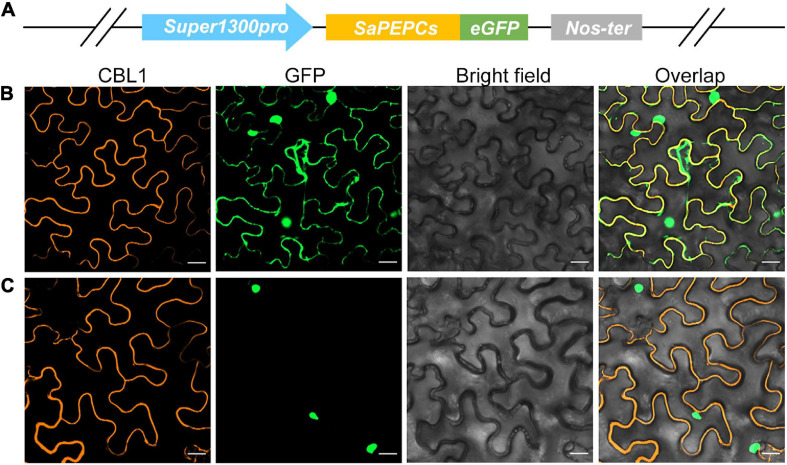
Subcellular localization of two types of SaPEPC in tobacco epidermal cells. **(A)** Schematic diagram of vector construction; **(B)** SaPEPC-1; **(C)** SaPEPC-2. CBL1, calcineurin B-like protein 1, membrane marker control; GFP, green fluorescent protein. Bar = 20 μm.

The prediction of *in silico* subcellular localization showed that all three SaPEPC proteins were most probably localized in the cytoplasm, with an average possibility of 24.1% in the nucleus (Table 1). Our transient transformation assay in tobacco epidermal cells showed that SaPEPC-1 had a strong fluorescence signal in the cytoplasm, plasma membrane, and nucleus (Figure 3B) while the fluorescence signal of SaPEPC-2 was mainly observed in the nucleus (Figure 3C and Supplementary Figure 4), which is consistent with the prediction by the Plant-mPLoc and YLoc^+^ software.

### Analysis of the *Cis*-Regulatory Elements and Activity of *SaPEPC* Promoters

**FIGURE 4:**
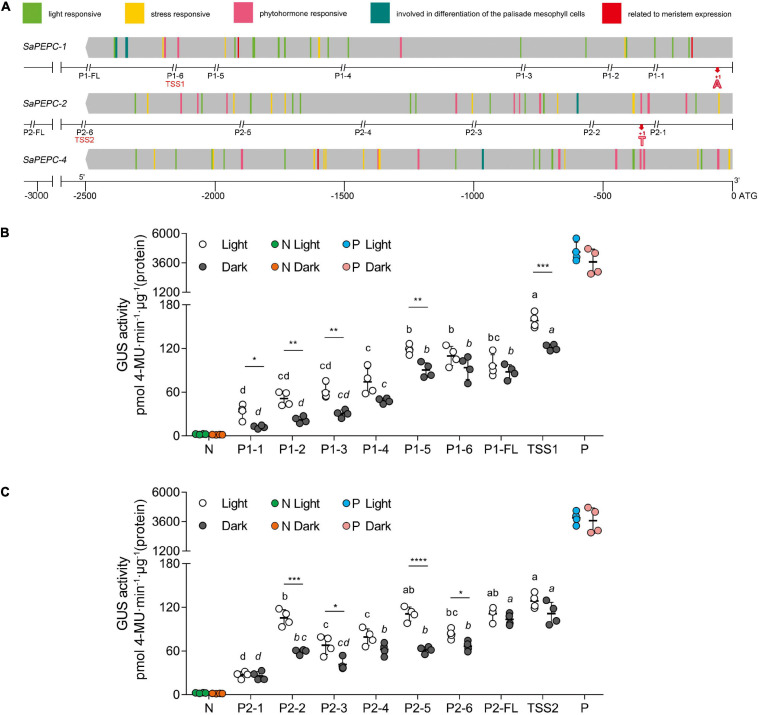
Sequence and activity analysis of *SaPEPC* promoters. **(A)** Predicted *cis*-elements on promoters of three *SaPEPC* genes and a schematic diagram of truncated sites of two *SaPEPC* promoters; **(B)** β-glucuronidase (GUS) enzyme activity driven by *SaPEPC-1* promoter; **(C)** GUS enzyme activity driven by *SaPEPC-2* promoter. The promoter regions (2,500 bp) of three *SaPEPC* genes were analyzed, and different *cis*-elements were present in color boxes. The numbers at the bottom of figure **(A)** indicate the distance to the start codon ATG. P1 and P2 represent *SaPEPC-1* and *SaPEPC-2* promoter, respectively. FL represents a full-length sequence from ATG of each promoter, which was consequently truncated into six fragments. The double italic slash represents the truncated site; the numbers 1–6 represent different truncated fragments ranking from the smallest to the largest. The red bold “A” (+1) and “T” (+1) marked the transcription start sites (TSSs), TSS1 and TSS2 represent a upstream sequence from TSSs of *SaPEPC-1* and *SaPEPC-2* promoters, respectively. N, negative control, injected with *A. tumefaciens* EHA105; P, positive control, injected with *A. tumefaciens* EHA105 harboring with pBI121 plasmid. Different lowercase letters indicate a significant difference between different fragments; *, **, ***, ****: Represent a significant difference between light and dark treatments of the same fragment at 0.05, 0.01, 0.001, 0.0001 level, respectively.

The retrieved 2,500 bp sequences upstream of the start codon of *SaPEPC* genes were queried to the PlantCARE database for a *cis*-regulatory element prediction. A total of 98 *cis*-elements were detected from the three *SaPEPC* genes, in addition to the phytohormone and specific expression-related *cis*-elements, the other two *cis*-elements were involved in palisade mesophyll cell differentiation and four (ARE, WUN-motif, LTR, and MBS) in response to abiotic stresses. In particular, light-responsive *cis*-elements were up to 12 varieties (i.e., AE-box, AT1-motif, ATCT-motif, Box-4, chs-CMA1a, G-box, GA-motif, GT1-motif, I-box, MRE, TCCC-motif, and TCT-motif) (Figure 4A and Supplementary Table 7), suggesting that *SaPEPCs* may largely be involved in light regulation. Further analysis of these results might help in understanding the role of *SaPEPC* genes in development, photosynthesis, and response to stresses.

Two *SaPEPC* promoters driving *GUS* expression *in vitro* revealed that the GUS activity was gradually decreased with the progressive 5′-flanking deletion of the upstream sequence (*F*_7,24_ = 46.26 and 42.69, *p* < 0.0001 for *SaPEPC-1* and *SaPEPC-2* under light, respectively; Welch’s *F*_7,10__.03_ = 263.99 and Welch’s *F*_7,10__.14_ = 32.85, *p* < 0.0001 for *SaPEPC-1* and *SaPEPC-2* under darkness, respectively) and significantly responded to light (e.g., *t*_6_ = 6.748, *p* = 0.0005 for TSS1 and *t*_6_ = 9.255, *p* < 0.0001 for P2-5, respectively) (Figures 4B,C). Interestingly, the upstream sequence starting from the TSS (named these segments as TSS1 and TSS2 for *SaPEPC-1* and *SaPEPC-2*, respectively, which were lacking the 5′-UTR sequence compared with P1-6 and P2-6 fragments) significantly promoted GUS activity compared to that of P1-6 fragment of *SaPEPC-1* (*t*_6_ = 5.848, *p* = 0.0011 under light; *t*_6_ = 3.343, *p* = 0.0155 under darkness) or P2-6 fragment of *SaPEPC-2* (*t*_6_ = 7.426, *p* = 0.0003 under light; *t*_6_ = 5.310, *p* = 0.0018 under darkness), respectively, suggesting that a 5′-UTR region may apply a repressive effect on the activity of the *SaPEPC* promoter (Supplementary Figure 1). Further analysis revealed that multiple stem-loop structures might be formed at the RNA level in the 5′-UTR sequence of *SaPEPCs*, that of *SaPEPC-2* presented a more complicated secondary structure with a folding free energy (Δ*G*) of −51.62 kcal mol^–1^ (Supplementary Figure 5). These facts imply that the 5′-UTR sequence of *SaPEPCs* may play an important regulatory role in gene expression.

### Spatial and Temporal Expression Patterns of *SaPEPC* Genes

**FIGURE 5:**
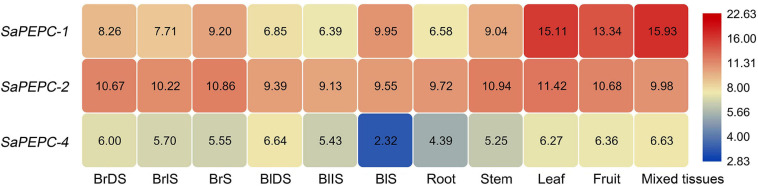
RNA sequencing (RNA-Seq) expression analysis of the three *SaPEPC* genes obtained from the public transcriptomic database of *S. aralocaspica*. BrDS, brown dry seed; BrIS, brown imbibed seed (imbibition for 1 h); BrS, seedling germinated from brown seed (imbibition for 24 h); BlDS, black dry seed; BlIS, black imbibed seed (imbibition for 24 h); BlS, seedling germinated from black seed (germination for 10 days). The fragments per kilobase of exon per million mapped reads (FPKM) values from the RNA-Seq data were log^2^ transformed.

**FIGURE 6:**
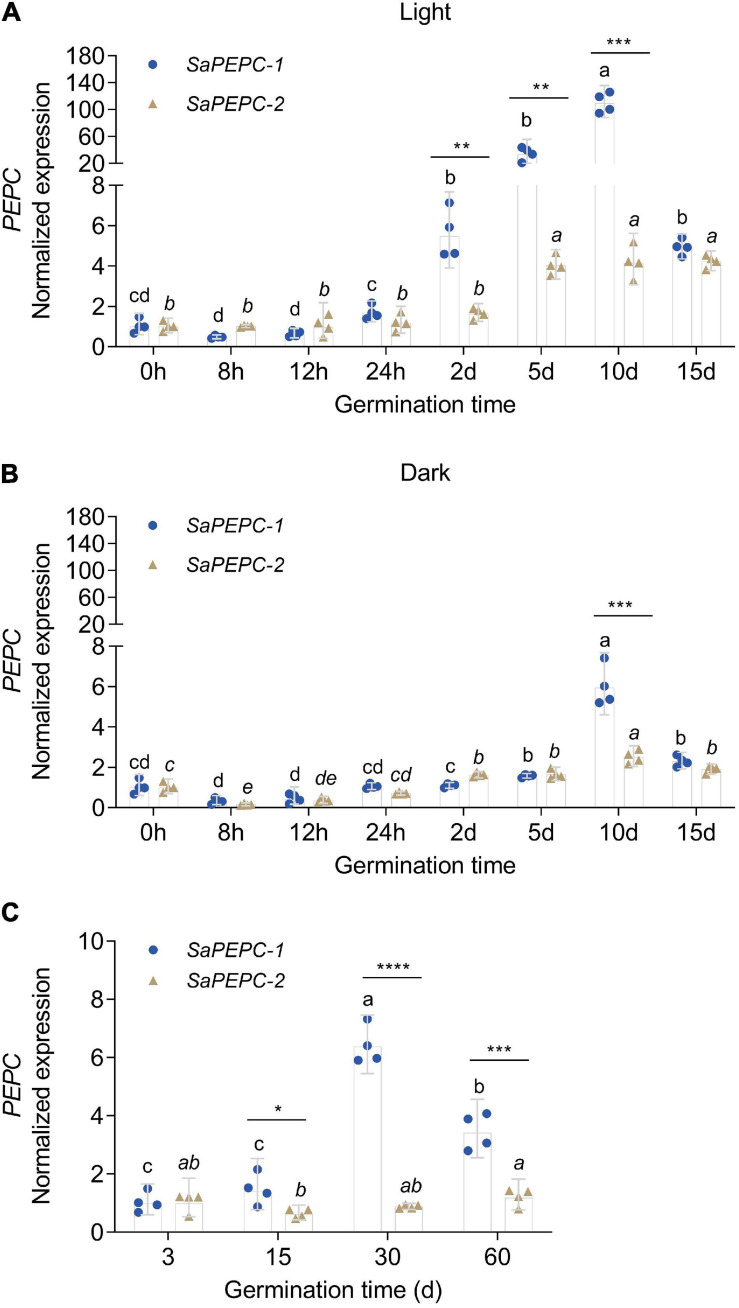
Relative expression of two *SaPEPC* genes in seed germination and the seedling growth of *S. aralocaspica*. **(A,B)** Seed germination under light or in the dark; **(C)** Seedling growth under light. Different lowercase letters indicate a significant difference of the same gene at different time points; *, **, ***, ****: Indicate a significant difference between *SaPEPC-1* and *SaPEPC-2* at the same time point at 0.05, 0.01, 0.001, 0.0001 level, respectively. Values are geometric mean ± 95% CI of four biological replicates.

Based on the available RNA-Seq data (Wang et al., 2017, 2019), the temporal expression patterns of three *SaPEPC* genes in dry, imbibed, and germinated seeds were analyzed by using the dimorphic seeds. *SaPEPC-1* accumulated more transcripts with the germination progression, and the brown seedlings responded quicker than the black ones; the transcripts of *SaPEPC-2* were accumulated to remain relatively constant and abundant in dimorphic seeds and seedlings while *SaPEPC-4* was expressed at lower levels in germination and both types of seeds (Figure 5). In our previous study, the transcriptional expression patterns of *SaPEPC-1* and *SaPEPC-2* were compared in terms of seed germination (0, 5, 10, and 15 days), different tissues (radicle, hypocotyl, and cotyledon), and salt stress (0, 100, 300, and 500 mmol⋅L^–1^) in dimorphic seeds under normal conditions (Cheng et al., 2016). In this study, we strengthened these data by emphasizing the effect of light/dark on the expression of two *SaPEPC* genes without distinguishing dimorphic seeds, and a much longer developmental period (i.e., 0 h, 8 h, 12 h, 24 h, 2 days, 3 days, 5 days, 10 days, 15 days, 30 days, and 60 days), more different tissue types (radicles, hypocotyls, cotyledons, and roots, stems, leaves of 15- and 90-day plants), and more different types of abiotic stresses (NaCl, mannitol, ABA, and light intensity) were applied. In developing seedlings and adult plants from brown seeds, the transcript accumulation of two genes was increased gradually with seedling growth, and reached the highest value at 10 days after germination and 30 days after emergence, respectively (Figure 6). Light or darkness treatment in the germination stage revealed that *SaPEPC-1* expression was more sensitive to light, and the transcript copies were approximately 20 times more than that in the dark on the 10th day of germination (Welch’s *t*_3__.028_ = 14.01, *p* = 0.0008) (Figures 6A,B). The results are consistent with our previous study (Cheng et al., 2016).

**FIGURE 7:**
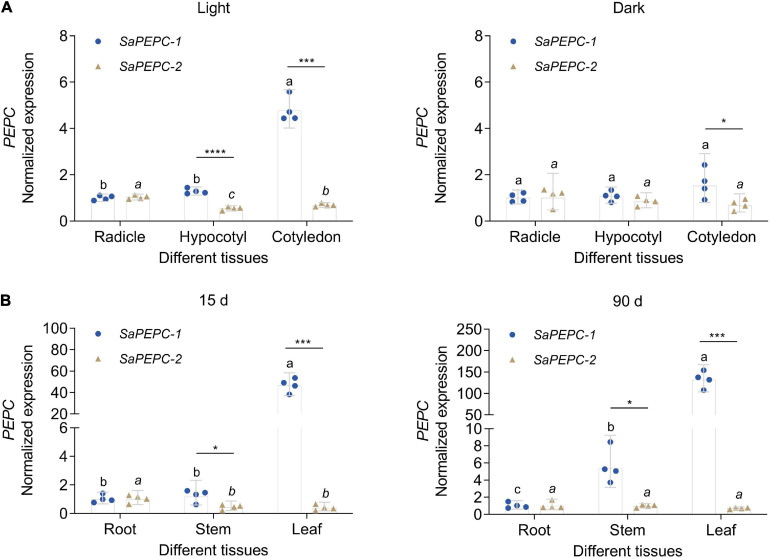
Relative expression of two *SaPEPC* genes in different tissues of *S. aralocaspica*. **(A)** Light and darkness treatments in seed germination; **(B)** Different growth stages of seedlings. Different lowercase letters indicate significant differences of the same gene in different tissues; *, ***, ****: Indicate a significant difference between *SaPEPC-1* and *SaPEPC-2* in the same tissue at 0.05, 0.001, 0.0001 level, respectively. Values are geometric mean ± 95% CI of four biological replicates.

The spatial expression patterns of *SaPEPC* genes according to the available data showed that *SaPEPC-2* was widely expressed in different tissues (root, stem, leaf, and fruit), *SaPEPC-1* was preferentially expressed in leaves and fruits, with lower expression in roots while the expression of *SaPEPC-4* was lower in all the tested tissues (Figure 5). With the help of qRT-PCR analysis, the expression patterns of *SaPEPC-1* and *SaPEPC-2* in different tissues were validated, partial results were consistent with the RNA-Seq data and our previous result (Cheng et al., 2016). In general, the accumulation of *SaPEPC-1* transcripts was significantly higher than that of *SaPEPC-2* in different tissues, especially in cotyledons (Welch’s *t*_3__.089_ = 15.03, *p* = 0.0005 under light and *t*_6_ = 2.709, *p* = 0.0351 under darkness) and leaves (Welch’s *t*_3__.005_ = 14.40, *p* = 0.0007 for 15-day seedling and Welch’s *t*_3__.000_ = 13.74, *p* = 0.0008 for 90-day-adult plant). In comparison with the high level in leaves, *SaPEPC-1* transcripts were hardly detected in developing roots and stems, whereas the *SaPEPC-2* expressed relatively higher in roots compared to other tissue types (Figure 7). These results suggest that *SaPEPC-1* and *SaPEPC-2* may play diverse biological functions in plant developmental processes.

### Expression Profiles of *SaPEPC* Genes in Response to Light and Abiotic Stresses

**FIGURE 8:**
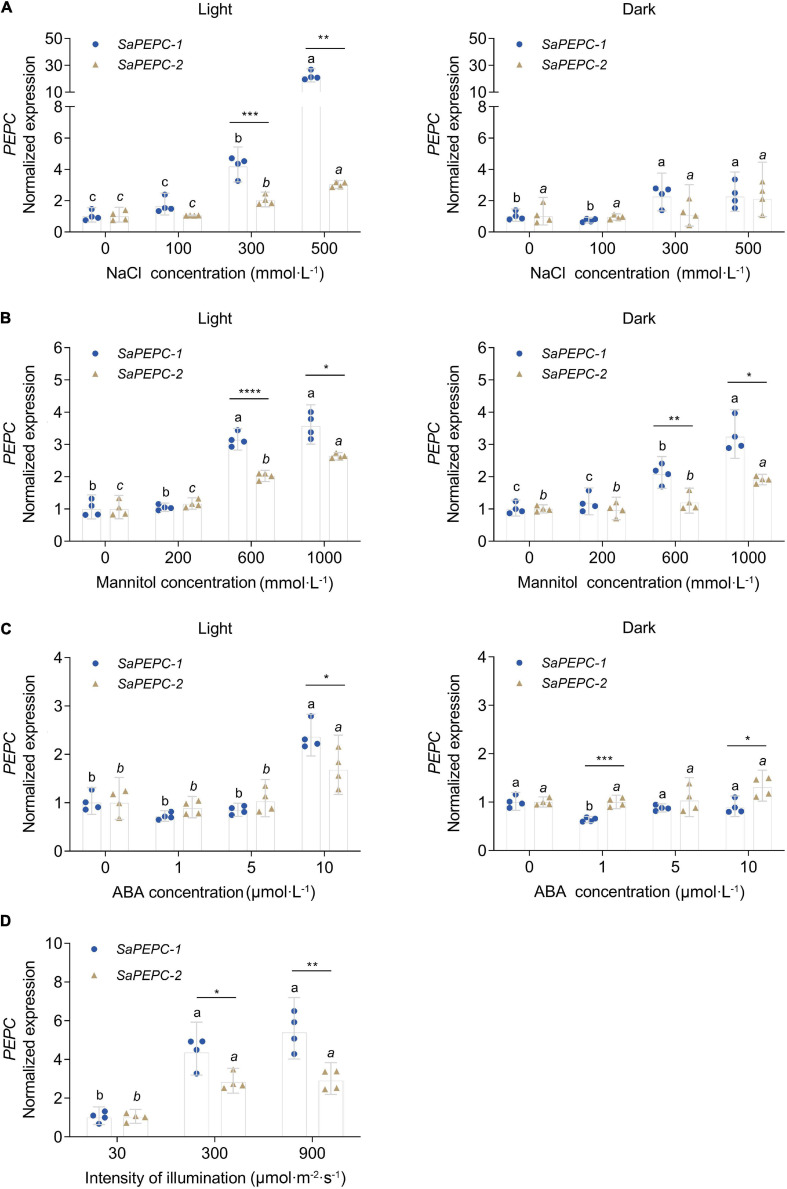
Relative expression of two *SaPEPC* genes under different light intensity and abiotic stresses. **(A–C)** Salt, drought, ABA stresses under light and darkness; **(D)** Treatment under different light intensity. Different lowercase letters indicate a significant difference of the same gene at different concentrations (conc.) or illumination; *, **, ***, ****: Indicate a significant difference between *SaPEPC-1* and *SaPEPC-2* at the same concentration or illumination at 0.05, 0.01, 0.001, 0.0001 level, respectively. Values are geometric mean ± 95% CI of four biological replicates.

To investigate the responses of *SaPEPCs* to NaCl, mannitol, ABA, and different light intensities in developing seedlings, the transcriptional expression patterns of *SaPEPC-1* and *SaPEPC-2* were analyzed. The results showed that two *SaPEPCs* were significantly upregulated by increasing the light intensity (*F*_2,9_ = 39.64, *p* < 0.0001 for *SaPEPC-1* and *F*_2,9_ = 28.99, *p* = 0.0001 for *SaPEPC-2*), especially under 300 (*t*_6_ = 3.500, *p* = 0.0128) and 900 μmol⋅m^–2^⋅s^–1^ (*t*_6_ = 4.586, *p* = 0.0037), the expression level of *SaPEPC-1* was significantly higher than that of *SaPEPC-2* (Figure 8D). Under various abiotic stresses, both *SaPEPCs* exhibited similarly positive responses to light or darkness while the transcript copies of *SaPEPC-1* were significantly higher than that of *SaPEPC-2* when exposed to 300 mmol⋅L^–1^ NaCl (*t*_6_ = 6.082, *p* = 0.0009), 600 mmol⋅L^–1^ mannitol (*t*_6_ = 9.432, *p* < 0.0001), and 10 μmol⋅L^–1^ ABA (*t*_6_ = 2.805, *p* = 0.0309) treatments under normal light conditions (Figures 8A–C).

### PEPC Enzyme Activity in Response to Development, Light, and Abiotic Stresses

**FIGURE 9:**
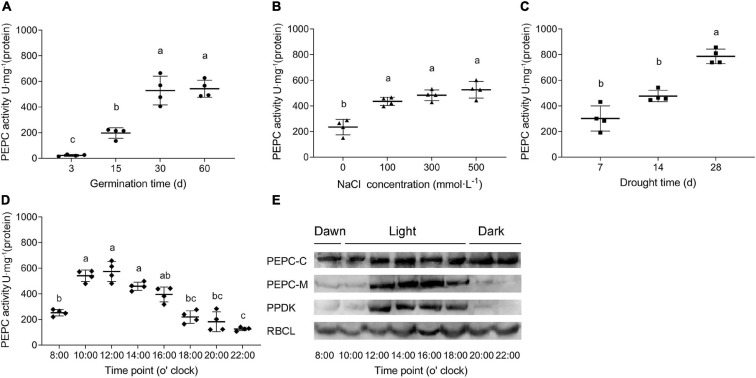
PEPC activity in *S. aralocaspica* leaves under various treatments. **(A)** Different developmental stages of seedlings; **(B)** NaCl stress; **(C)** Natural drought stress (days of withholding water); **(D)** Activity variations within a whole day; **(E)** Variations of protein amounts of key photosynthetic enzymes. PEPC-C, total PEPC; PEPC-M, phosphorylated PEPC; RBCL, Rubisco large subunit. Different lowercase letters indicate significant difference at different treatments. Values are means ± SD of four replicates.

Enzyme activity of SaPEPCs was measured in the leaves of different developmental stages and under different light intensities as well as abiotic stresses. As shown in Figures 9A–C, PEPC activity varied in consistency with the expression pattern of *SaPEPC* genes, which was significantly increased with the germination time extension (Welch’s *F*_3,5__.152_ = 101.5, *p* < 0.0001) and the stress enhancement (*F*_3,12_ = 24.91, *p* < 0.0001 for salt stress and *F*_2,9_ = 48.49, *p* < 0.0001 for drought stress). The diurnal variation in light intensity resulted in a gradual increase of PEPC activity in the morning, and reached the highest value (average 574.44 U⋅mg^–1^ protein) at 12:00, then dramatically decreased thereafter until 22:00 (Welch’s *F*_7,9__.908_ = 75.16, *p* < 0.0001), appeared as a “unimodal” curve (Figure 9D). The protein accumulation of representative photosynthetic enzymes [total PEPC (PEPC-C), phosphorylated PEPC (PEPC-M), PPDK, and RBCL] was also analyzed by the varying light intensity from the morning to the evening (Figure 9E). Among them, the amount of RBCL appeared to be abundant and relatively constant; PEPC-C increased with time elongation and reached the highest level from 12:00 onward; PEPC-M and PPDK were remarkably accumulated from 12:00 to 18:00 (the period with the highest light intensity in a day), and decreased significantly from then on. Our results showed that these proteins could apparently be induced by increasing light intensity, especially PEPC phosphorylation, such an expression pattern was corresponding to the diurnal changes of PEPC enzyme activity, suggesting a close relationship between light intensity and PEPC phosphorylation, and the consequent PEPC activity.

### Validation of SaPEPCs in Abiotic Stress Tolerance in *E. coli*

**FIGURE 10:**
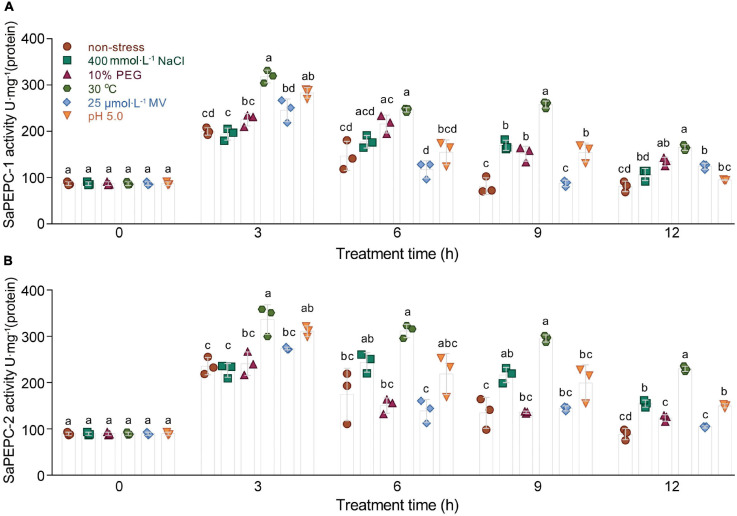
Ectopic expression of two *SaPEPC* genes confers abiotic stress tolerance to *Escherichia coli*. **(A,B)** Activity analysis of recombinant SaPEPC-1 and SaPEPC-2 under abiotic stresses [400 mmol⋅L^– 1^ NaCl, 10% PEG, 25 μmol⋅L^– 1^ methyl viologen (MV), 30°C and pH 5.0]. Different lowercase letters indicate significant difference among different stresses at the same time. Values are means ± SD of three replicates.

Enzyme activity was further determined by the ectopic expression of *SaPEPCs* in *E. coli* under salt (NaCl), drought (PEG 6000), oxidation (MV), temperature, and acid/base (pH) stresses. SaPEPC-1 and SaPEPC-2 proteins were resolved by the SDS-PAGE and detected by an immunoblot analysis, which revealed a recombinant protein with the MW of 110 kDa in accordance with the theoretical values. SaPEPC-1 was expressed in the supernatant and inclusion bodies while SaPEPC-2 was mainly expressed in the inclusion bodies of the cells (Supplementary Figure 6). In our previous study, due to the lack of complete coding sequence of *SaPEPC-2*, only *SaPEPC-1* recombinant strain was analyzed with the time course of growth and enzyme activity [under 400 mmol⋅L^–1^ NaCl, 10% (w/v) PEG, 25°C, 75 μmol⋅L^–1^ MV, and pH 5.0] (Cheng et al., 2016). In this study, we further supplemented the corresponding data of *SaPEPC-2* recombinant strain, our results showed that the overexpression of *SaPEPC-2* could also significantly enhance cell growth and enzyme activity under different stress conditions, except for the cases of growth at 25°C and the enzyme activity under 75 μmol⋅L^–1^ MV (compared with *SaPEPC-1* recombinant strain) (Supplementary Figures 7, 8). Based on the abovementioned analysis, the enzyme activity of both *SaPEPC* recombinant strains was determined simultaneously under the conditions of 400 mmol⋅L^–1^ NaCl, 10% (w/v) PEG, 30°C, 25 μmol⋅L^–1^ MV, and pH 5.0. With an increase in stress time (to a total of 12 h), the PEPC activity of two strains increased significantly and reached the maximum value at 3 h (Welch’s *F*_4,34__.42_ = 69.53, *p* < 0.0001 for SaPEPC-1 and Welch’s *F*_4,34__.21_ = 95.82, *p* < 0.0001 for SaPEPC-2), and then reduced but generally remained higher than that of the control (Figure 10). In general, the SaPEPC-2 activity was higher than that of SaPEPC-1.

### Carboxylase Activity and Influence of Different Effectors on Enzyme Kinetics

**FIGURE 11:**
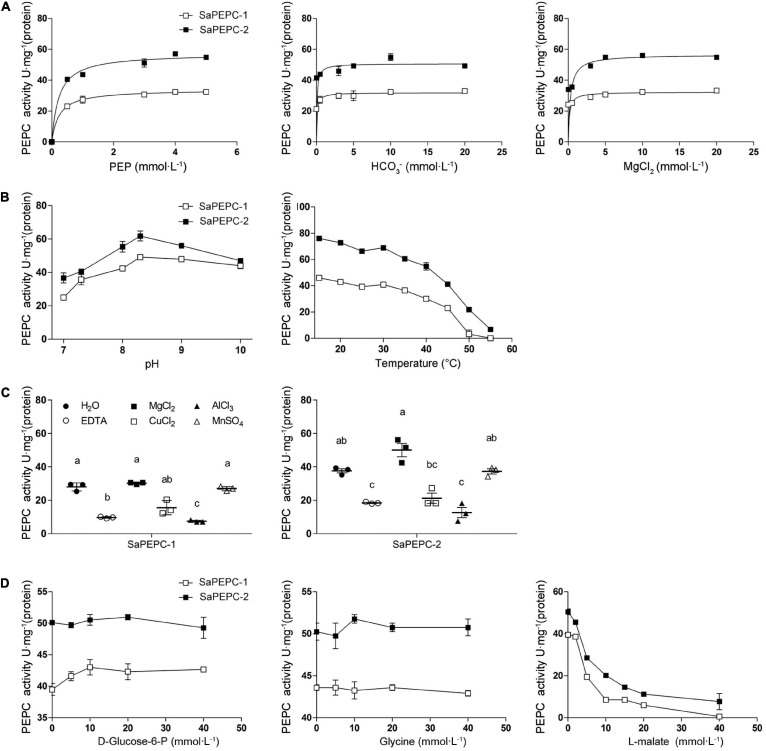
Effects of different factors on enzyme activity of purified recombinant SaPEPC proteins. **(A)** PEP, HCO_3_^–^, and MgCl_2_ saturation curves; **(B)** Test of pH and temperature stability; **(C)** Test of metal ion stability; **(D)** Test of allosteric activator and inhibitor stability. Different lowercase letters indicate significant difference among different metal ion treatments. Values are means ± SD of three replicates.

Different conc. of PEP, Mg^2+^, and HCO_3_^–^ were applied in the measurement of the recombinant SaPEPC activity to assess their effects. At pH 8.0 and 25°C, the highest *K*_m_ and *V*_max_ values of SaPEPC-1 or SaPEPC-2 were estimated to be approximately 0.237 or 0.231 mmol⋅L^–1^ and 33.85 or 57.32 U⋅mg^–1^ protein, respectively, with different conc. of PEP. On the contrary, the lowest *K*_m_ and *V*_max_ values were present under different conc. of HCO_3_^–^, the saturation conc. was quickly reached, and the catalytic efficiency (*K*_cat_/*K*_m_) was 39.33 × 10^3^ or 66.35 × 10^3^ (mmol⋅L^–1^)^–1^min^–1^, respectively, which was two times higher than that with PEP and MgCl_2_. Moreover, the catalytic efficiency of SaPEPC-2 with PEP or HCO_3_^–^ was about two times as much as that of SaPEPC-1, indicating that SaPEPC-2 may possess a higher substrate affinity compared to SaPEPC-1 (Table 2 and Figure 11A).

**TABLE 2:** Enzyme activity of purified SaPEPC proteins and enzymatic kinetics parameters of PEP, HCO_3_^–^ and MgCl_2_.

Protein	Specific activity (U⋅mg^−1^)	Enzymatic kinetics parameters											
		PEP				HCO_3_^–^				MgCl_2_			
		*V*_max_ (U⋅mg^−1^)	*K*_m_ (mmol⋅L^−1^)	*K*_cat_ (min^–1^)	*K*_cat_/*K*_m_ [(mmol⋅L^−1^)^–1^ min^–1^]	*V*_max_ (U⋅mg^−1^)	*K*_m_ (mmol⋅L^−1^)	*K*_cat_ (min^–1^)	*K*_cat_/*K*_m_ [(mmol⋅L^−1^)^–1^ min^–1^]	*V*_max_ (U⋅mg^−1^)	*K*_m_ (mmol⋅L^−1^)	*K*_cat_ (min^–1^)	*K*_*cat*_/*K*_m_ [(mmol⋅L^−1^)^–1^ min^–1^]
SaPEPC-1	32.437 ± 1.21	33.85 ± 0.76	0.237 ± 0.036	3.76 × 10^3^	15.86 × 10^3^	31.87 ± 3.21	0.090 ± 0.15	3.54 × 10^3^	39.33 × 10^3^	32.26 ± 3.88	0.149 ± 0.18	3.58 × 10^3^	24.03 × 10^3^
SaPEPC-2	54.927 ± 3.17	57.32 ± 1.25	0.231 ± 0.032	6.37 × 10^3^	27.57 × 10^3^	50.73 ± 6.06	0.085 ± 0.17	5.64 × 10^3^	66.35 × 10^3^	56.59 ± 6.04	0.301 ± 0.25	6.29 × 10^3^	20.90 × 10^3^

*The reaction conditions are pH 8.0, 25°C. The data are represented as means with standard deviations from three independent experiments.*

The influence of various effectors on the stability of SaPEPC-1 and SaPEPC-2 activities was investigated. The enzymatic activity of two SaPEPCs remained relatively constant at a range of temperature from 15 to 40°C while declining rapidly from 40 to 55°C, and could almost not be detected at 55°C and above, between them, SaPEPC-2 was able to tolerate higher temperature than that of SaPEPC-1. The pH stability test showed that both SaPEPCs presented the highest activity at pH 8.3, whereas the activity decreased significantly when the pH value was less than 8.0 (Figure 11B). Al^3+^ ion showed a significant inhibition to the enzyme activity of two SaPEPCs, which was similar to the effect of EDTA (metal ion-chelating agent). When exposed to Cu^2+^ ion, only approximately 50% activity of both SaPEPCs was detected compared to the control. As the metal ion cofactors of PEPC, Mg^2+^, or Mn^2+^ ions displayed the highest activity and had no significant difference compared to the control (Figure 11C). The kinetics of the recombinant enzymes were examined in the presence of allosteric activators and inhibitors under standard conditions. As shown in Figure 11D, with an increase in the concentration of glucose-6-phosphate and glycine, the activity of both SaPEPCs showed a slight increase while L-malate significantly inhibited their activity.

## Discussion

Phosphoenolpyruvate carboxylase plays pivotal roles in the carbon fixation of photosynthesis and a variety of metabolic and stress pathways. Therefore, clarifying different *PEPC* isoforms and their properties is necessary for further understanding of their functions. *S. aralocaspica* has evolved a unique SC C_4_ pathway (Edwards et al., 2004). As a key photosynthetic enzyme, PEPC has been studied on the light-regulatory characteristics, enzyme activity, responses to multiple stresses, etc., in *S. aralocaspica* (Lara et al., 2006; Cheng et al., 2016; Koteyeva et al., 2016). However, a genome-wide analysis and the comparative study on different PEPC isoforms in *S. aralocaspica* have not been well documented. In this study, we characterized a new BTPC (SaPEPC-4), which presented a lower expression level in germination and all tested tissues compared with other isoforms. In addition to the two PTPCs (SaPEPC-1 and SaPEPC-2) we previously reported, three members of the *PEPC* gene family in *S. aralocaspica* have been classified so far. With the achievement of the complete coding sequences of the two PTPC genes, the comparative study was conducted and the results showed that *SaPEPC-1* and *SaPEPC-2* presented different spatiotemporal expression patterns and distinct subcellular localizations. Compared to *SaPEPC-2, SaPEPC-1* was much more active in the progression of plant development and in response to various stresses. *SaPEPC-1* was especially more responsive to light variations in comparison with *SaPEPC-2* (Figures 6–8), which may be partially related to the *cis*-elements distributed on the promoter. The more complicated secondary structure and higher free energy of the 5′-UTR sequence found in *SaPEPC-2* might apply a repressive effect on expression *in vivo*. The expression trend of two SaPTPCs in response to light and abiotic stresses was matched with the PEPC activity in *S. aralocaspica*. The recombinant SaPEPC-2 showed a higher enzymatic activity than SaPEPC-1 with different effectors *in vitro*, and both exhibited a similar pattern in response to various stresses when ectopic expressed in *E. coli*, which might be attributed to being driven by the same T7 RNA polymerase gene promoter (Dubendorff and Studier, 1991). So far, for the limited comparative study on different PEPCs, it is still uncertain whether PEPCs of different plant-type isoforms or from different plants will achieve the same response or not. Our results suggest that *SaPEPC-1* may play a major role in the C_4_ photosynthetic pathway in *S. aralocaspica*.

In this study, a genome-wide analysis identified the third *PEPC* isoform from the available genomic data of *S. aralocaspica* (Wang et al., 2019). Only three *PEPC* genes were found so far in *S. aralocaspica*, which were less than that in other plant species, such as *Z. mays* (6) and *G. max* (10) (Supplementary Table 4), and which was only one half of the numbers in *C. quinoa*, an allotetraploid species in Chenopodiaceae (Yasui et al., 2016). Similarly, *PEPC* gene number in tetraploid cotton species is about two times as that in diploid cottons, suggesting that the difference of *PEPC* number may be associated with the interspecific hybridization or whole-genome duplication events (Wang et al., 2016; Zhao et al., 2019). PTPC and BTPC are significantly different in gene sequence and molecular structure (O’Leary et al., 2011b). In this study, the newly identified SaPEPC-4 (belonging to the BTPC subfamily) exhibited a more complicated gene structure, e.g., with 20 exons, which are much more than that in PTPCs (Figure 2). The most conserved 10 motifs predicted in 135 PEPCs from 27 different species were all found in the three SaPEPCs, suggesting the common origins and the evolutionary patterns of PEPCs in Viridiplantae (Supplementary Figure 3). Notably, however, significant discrepancies on the introns and UTR regions usually exist among various *PEPC* genes, e.g., the third intron of *SaPEPC-2* was about 10 times longer than *SaPEPC-1* in *S. aralocaspica* (Figure 2 and Supplementary Figure 2). Larger introns may have more mutable sites to increase genetic diversity and promote gene evolution through alternative splicing (Kandul and Noor, 2009), whereas genes with shorter introns are selectively favored to reduce the costs of transcription and tend to be highly expressed (Seoighe et al., 2005). In the evolutionary process of multi-gene families, the diversification of gene structure may result in new functions of the gene in adaptation to the changes in the environment. However, the significance of a striking difference in the size of the third intron between *SaPEPC-1* and *SaPEPC-2* still needs to be interpreted.

All plant genomes sequenced to date contain at least one BTPC gene, including the gene of ancestral green algae (O’Leary et al., 2011a), however, the role of BTPC in plant cells remains limited. In developing castor oil seeds, BTPC functions as a catalytic and regulatory subunit appeared to interact with PTPC to form a stable Class-2 PEPC complex, which may facilitate the rapid refixation of respiratory CO_2_ to replenish the C-skeletons of tricarboxylic acid cycle (TCA cycle) (O’Leary et al., 2009; Park et al., 2012). In the male gametophyte of *Lilium Longiflorum*, BTPC forms a complex with PTPC and monoubiquitinated PTPC to accelerate the accumulation of storage substances during pollen maturation (Igawa et al., 2010). In *Arabidopsis*, the downregulation of BTPC gene might increase the expression of the other three PTPC genes, indicating a transcriptional interaction between BTPC and PTPC in vascular plants (Wang et al., 2012). In this study, the transcriptional levels of three *SaPEPC* genes in *S. aralocaspica* were analyzed based on the database. Considering its detection in five tissues and at different developmental stages of the dimorphic seeds, *SaPEPC-4* (BTPC) gene showed a lower expression level in all combinations (Figure 5), which is consistent with that in *Arabidopsis*, soybean, and foxtail millet (Sánchez and Cejudo, 2003; Wang et al., 2016; Zhao et al., 2020). So far, however, we have not achieved the FL cDNA sequence of *SaPEPC-4* according to the genomic sequence published recently in *S. aralocaspica* (Wang et al., 2019). Therefore, further studies are needed to characterize the *SaPEPC-4* gene and verify its involvement in plant metabolism in *S. aralocaspica*.

The comparative study of two PTPC genes (*SaPEPC-1* and *SaPEPC-2*) was performed (for the lack of the complete coding sequence of *SaPEPC-4*), and they presented different subcellular localization and different expression patterns (Figures 3, 5). PEPCs are ubiquitous cytosolic enzymes in higher plants, e.g., tomato SlPEPC1, SlPEPC2, and SlPEPC3 (Waseem and Ahmad, 2019), except for rice Osppc4 targeting to the chloroplast (Chollet et al., 1996; Masumoto et al., 2010). In *S. aralocaspica*, the immunolabeling of PEPC-C protein in the fully mature chlorenchyma cells showed an even distribution throughout the cytoplasm (Koteyeva et al., 2016). In this study, SaPEPC-1 exhibited a strong fluorescent signal in the cytoplasm, whereas SaPEPC-2 appeared to exhibit nuclear localization in tobacco epidermal cells. For the potential disadvantage of any transient expression system, i.e., the overexpression or the saturation of the protein may alter the subcellular distribution (Sparkes et al., 2006), especially with the strikingly different background of a SC C_4_ photosynthesis system, it is more necessary to employ a similar singular chlorenchyma cell system to determine the subcellular localization of different PEPCs. The protoplast system for the transient gene expression from the chlorenchyma cells of *B. sinuspersici* (another SC C_4_ species) has been established (Lung et al., 2011), which should be a reliable system for the determination of the subcellular localization of the PEPC isoforms of SC C_4_ species in the future.

Photosynthetic PEPC is highly expressed in C_4_ plant leaves, whereas non-photosynthetic PEPC may have no expression specificity (Westhoff and Gowik, 2004). In our previous study, the expression level of *SaPEPC-1* was significantly higher than that of *SaPEPC-2* in cotyledons, and with seed germination progression (from dry seed to germination for 15 days) (Cheng et al., 2016). In this study, by emphasizing the effects of light/darkness and the extended developmental period (from seed germination to a 90-day adult plant), we found that *SaPEPC-1* was mainly expressed in chlorenchyma tissues (cotyledons and leaves), in which light and progressive development significantly induced its expression (Figures 6, 7, 8D), our results are consistent with that of C_4_-type *PEPC* described in sorghum and maize (Crétin et al., 1991; Schäffner and Sheen, 1992). However, the expression level of *SaPEPC-2* altered in a limited range with an increase in light intensity, progressive development, and tested tissues, which is more like the *ppc-aL2* – a housekeeping gene isoform of *PEPC* in sugarcane (Besnard et al., 2003). The performance of *SaPEPC-1* was similar to *Arabidopsis AtPPC2*, which is the only *PEPC* gene expressed in green tissues and participates in carbon fixation in C_3_ plants (Li et al., 2014; You et al., 2020), and both belong to the PTPC IV subgroup (Figure 1). In our previous study, *SaPEPC-1* shared a high homology with the *PEPC* members of C_4_ species and was clustered into C_4_ clade while *SaPEPC-2* was located in the C_3_ cluster (Cheng et al., 2016). According to Rosnow et al. (2014), C_4_ species commonly recruit *ppc-1* gene (an ortholog of *SaPEPC-1*) for use in C_4_ photosynthesis. Furthermore, with the supporting evidence of the strong light activation of *SaPEPC-1* expression and significantly increased PEPC activity with the progression of development (Figures 4B, 6C, 9A), all these clues suggest that SaPEPC-1 might be a C_4_-like PEPC isoform to participate in the C_4_ photosynthetic pathway in *S. aralocaspica*.

Phosphoenolpyruvate carboxylase participates in plant response to various stresses and hormone signal transduction (Zhao et al., 2019; Gallego-Tévar et al., 2020). In our previous study, both *SaPTPCs* showed a significant upregulation under salinity while *SaPEPC-1* accumulated much more transcripts than that of *SaPEPC-2* (Cheng et al., 2016). To explore their difference in transcriptional regulation, we examined their promoter sequences and found 15 varieties of stress-responsive *cis*-elements, surprisingly, *SaPEPC-2* contains about three times more stress- and hormone-responsive elements than *SaPEPC-1* (Figure 4A and Supplementary Table 7). Based on the predictions, the expression profiles of two *SaPTPCs* were further analyzed under the stresses of salt, drought, ABA, and high light intensity, the results indicated that both genes could positively respond to abiotic stresses, but the transcript abundance of *SaPEPC-1* was much greater than that of *SaPEPC-2* (Figure 8). In addition, the enzyme activity of recombinant SaPEPC-1 and SaPEPC-2 was simultaneously analyzed in comparison with the result of SaPEPC-1 only in Cheng et al. (2016), both of which were increased in response to various stresses, and consequently, the growth advantage of the recombinant strains was enhanced (Figure 10 and Supplementary Figure 7). The ectopic expression of peanut *AhPEPC2* may confer more osmotic stress resistance to the recombinant strains compared to that of *AhPEPC1* and *AhPEPC5* (Tu et al., 2021). The overexpression of pearl millet C_4_-specific *PEPC* in *E. coli* also displays a positive effect against abiotic stresses by increasing PEPC activity (Singh et al., 2012). It has been reported that salinity mainly applies osmotic stress on *E. coli* (Liu et al., 2019) and enhanced PEPC activity can catalyze the synthesis of malate, besides as an osmolyte and also to potentially regulate intracellular pH balance and counteract the excess toxic ions to help cells to tolerate stresses (Martinoia and Rentsch, 1994).

To further compare the difference between the two SaPTPC isoforms, the recombinant proteins of SaPEPC-1 and SaPEPC-2 were produced to analyze their enzymatic kinetics *in vitro*, which may avoid the effect of post-translational modification *in vivo* (Rao et al., 2008). Purified SaPEPC-1 and SaPEPC-2 exhibited a specific carboxylation enzymatic activity (32.437 and 54.927 U⋅mg^–1^ protein, respectively) (Table 2), which is comparable with the PEPC from other plant species and algae (ranging from ∼20 to 35 U⋅mg^–1^ protein) (Mamedov et al., 2005; Chang et al., 2014). PEPC activity is affected by substrates, ions, conc. of metabolites, activators/inhibitors, temperature, pH, etc., significant differences in enzymatic properties are observed among the different types and sources of PEPCs (Paulus et al., 2013). Similar to *Arabidopsis* AtPPC3, both SaPTPCs exhibited an optimal activity at pH 8.3 while SaPTPCs had a higher heat stability by retaining 50% activity at 45°C in the presence of a bivalent metal cofactor Mg^2+^ (Figures 11B,C), in comparison with AtPPC3 showing the loss of 90% activity (O’Leary et al., 2009). Glucose-6-phosphate and glycine are able to activate maize C_4_-PEPC activity by more than 2-fold, and its root-PEPC is more sensitive to the feedback inhibitor L-malate (Dong et al., 1998). In this study, only a slight increase in two SaPTPC activities was detected by applying activators while L-malate significantly inhibited their activity (approximately 4.6- and 2.5-fold for SaPEPC-1 and SaPEPC-2, respectively) (Figure 11D), which were similar to the non-C_4_ or root-type PEPC in maize. The HuPPC3 in pitaya is also more sensitive to malate but involved in the initial fixation of atmospheric CO_2_ in crassulacean acid metabolism (CAM) photosynthesis (Nomura et al., 2020). The catalytic efficiency values (*K*_cat_/*K*_m_, (mmol⋅L^–1^)^–1^min^–1^) for the substrates of PEP and HCO_3_^–^ of *E. coli* PEPC are 1.5 × 10^3^ and 9.0 × 10^4^, respectively (Kai et al., 1999; Lee et al., 2013). The *K*_cat_/*K*_m_ values of purified wild-type and N-terminal truncated PEPCs in *Phaeodactylum tricornutum* for PEP are about 2–4 times higher than that of *E. coli* PEPC, while for HCO_3_^–^ the values are about 15–34% (Chang et al., 2014). In this study, the *K*_cat_/*K*_m_ values of recombinant SaPEPC-1 and SaPEPC-2 for PEP were 10 and 18 times of that of *E. coli* PEPC, and the values for HCO_3_^–^ were only 44 and 74%, respectively (Table 2), suggesting that two SaPTPCs may be more efficient in CO_2_ utilization.

In this study, the *K*_m_ values of two recombinant SaPTPCs were lower compared to those native PEPCs in *S. aralocaspica* (Rosnow et al., 2015), which is also observed in *Pennisetum glaucum* (Singh et al., 2012). PTPCs commonly contain a serine residue at N-terminus, which can be phosphorylated by PEP carboxylase kinase (PPCK) and dephosphorylated by protein phosphatase 2A (PP2A) (O’Leary et al., 2011a). The higher *K*_m_ value of native PEPCs might be involved in the phosphorylation activation *in vivo*. In Kranz-type and SC C_4_ species, the phosphorylation of PEPC is triggered by light and consequently leads to an increase in catalytic activity of the enzyme (Ping et al., 2018). According to Lara et al. (2006), the phosphorylation of PEPC is enhanced by the increasing light intensity from 7:00 to 17:00, and dramatically reduced by the decreasing light intensity from 20:30 to 00:30, with the major proportion of PEPC-M at 13:00 and 17:00 in *S. aralocaspica*. In this study, the diurnal activity changes of different photosynthetic enzymes were further analyzed although the total amount of SaPEPC protein remained basically constant, the accumulation of phosphorylated SaPEPC and SaPPDK was initiated from dawn (8:00), reached the maximum value by midday (14:00, in winter), and drastically decreased before the sunset (20:00), the key period for PEPC phosphorylation occurred between 12:00 and 18:00, the dephosphorylation of SaPEPC was almost complete by night (22:00) (Figure 9E), which is in accordance with the observations by Lara et al. (2006), and in maize (Ueno et al., 2000; Fukayama et al., 2003), indicating that the higher light intensity stimulates PEPC phosphorylation. In correspondence to such changes, PEPC activity also fluctuated within a whole day and reached the maximum value at 12:00 in *S. aralocaspica* (Figure 9D). Not only did the light intensity enhance the PEPC activity, but also other factors could apply effects on, e.g., PEPC activity changed from a couple of tens to several hundred (U⋅mg^–1^ protein) with the progression of development and salt or drought stress strengthening (Figures 9A–C), which was also matched with *PEPC* gene expression trends at the transcriptional level (Figures 6, 8). Phosphorylated PEPC exhibits low malate sensitivity while the dephosphorylated PEPC is strongly inhibited by malate to avoid a futile photosynthetic cycle (Nomura et al., 2020). This might be a cue for the sensitive inhibition effect of malate on SaPEPC-1 activity *in vitro*. It is worth noting that, in our previous study, the two patterns of the daily variations of PEPC activity were present as a “double peak” (outdoor) or “unimodal” (greenhouse) in *S. aralocaspica* (Liu et al., 2020), which were closely associated with the cultivation conditions, e.g., illumination hours, temperature, etc. In this study, the plants were cultivated and analyzed in a greenhouse, so the PEPC activity presented a “unimodal” trend, which is also an evidence to support that PEPC activity varies with a change in the light intensity.

SaPEPC-1 recombinant protein presented lower activity and enzymatic kinetics compared to SaPEPC-2 (Table 2), while the transcriptional level of *SaPEPC-1* in *S. aralocaspica* was significantly higher than that of *SaPEPC-2* (Figures 6, 8). What a possible regulatory mechanism is behind the expression of these *SaPEPC*s? With the investigation of the promoter activity of *SaPEPC-1* and *SaPEPC-2* by driving *GUS* gene, we found no significant difference in the promoter activity at the transcriptional level *in vitro* (*t*_6_ = 1.655, *p* = 0.1491 under light and *t*_6_ = 2.415, *p* = 0.0522 under darkness) while two *SaPEPC* promoters similarly enhanced *GUS* expression at the translational level when the 5′-UTR sequence was deleted (Figures 4B,C), suggesting that two *SaPEPCs* might have experienced more complicated regulations *in vivo*, e.g., the posttranscriptional modulation by the 5′-UTR sequence. The secondary structures of 5′-UTR have been characterized as the negative regulators of gene expression at both transcriptional and translational levels (Stefanovic et al., 2000; Bunimov et al., 2007). In the tomato pollen-specific promoter of *LAT59* gene, the stem-loop structure of 5′-UTR dramatically decreased the messenger RNA (mRNA) accumulation of the reporter gene without affecting the translation rate and mRNA stability (Curie and McCormick, 1997). A more complicated 5′-UTR secondary structure is located upstream of nitrate reductase (NR) gene in *Chlorella vulgaris*, which accelerates the degradation of NR transcripts (Cannons and Cannon, 2002). In this study, the predicted secondary structure of the 5′-UTR of *SaPEPC-1* was relatively simple with less than −4 kcal/mol free energy (Δ*G*) while in *SaPEPC-2*, that was complicated and stable with a maximum Δ*G* value of −51.62 kcal/mol at the RNA level (Supplementary Figure 5). It is proposed that every −10 kcal/mol of Δ*G* is sufficient to reduce the translation efficiency by about 50%, and −50 kcal/mol of Δ*G* may inhibit more than 85% of the translation efficiency (McCarthy, 1998; Brunn et al., 2012). Therefore, the higher Δ*G* of 5′-UTR secondary structure might inhibit the *SaPEPC-2* expression *in vivo*; alternatively, *SaPEPC-2* might affect the stability of mRNA with the secondary structure of 5′-UTR, which consequently resulted in the abundance of a lower transcript in *S. aralocaspica*.

## Conclusion

This study characterized the members of a genome-wide *PEPC* gene family and comparatively analyzed the two PTPC isoforms in SC C_4_ species *S. aralocaspica.* With the *in silico* analysis of *S. aralocaspica* genomic database, a new bacterial-type *SaPEPC-4* gene was identified based on the previously reported two PTPC genes (*SaPEPC-1* and *SaPEPC-2*, the latter with a partial sequence), while its function is still unknown. Three *SaPEPC* genes were differentially expressed in roots, stems, leaves, fruits, and heteromorphic seed development, and presented distinct subcellular localization patterns. The transcript abundance and enzyme activity (native or recombinant) of the two PTPC genes (*SaPEPC-1* and *SaPEPC-2*) were stimulated by light and abiotic stresses. In *S. aralocaspica*, the transcript copies of *SaPEPC-1* were significantly higher than that of *SaPEPC-2* under various stresses, but the enzymatic kinetics (*V*_max_ and *K*_cat_/*K*_m_ for different substrates) and biochemical properties (heat stability, activator and inhibitor responses) of the latter were higher than that of the former *in vitro*. The 5′-UTR regions of the two *SaPEPC* promoters might apply the repression effect on the expression of *PEPC* genes at transcriptional, posttranscriptional, and/or translational levels. In terms of phylogenetic relationship, spatiotemporal expression pattern, light sensitivity, *SaPEPC-1* gene is more likely to be recruited as a C_4_-type PEPC; whereas *SaPEPC-2* behaves like a non-photosynthetic housekeeping gene. However, the details for the regulation of different *PEPC* genes *in vivo* still need to be interpreted with more efforts. Our findings may lead to decipher the exact roles of PEPC isoforms in C_4_ photosynthesis, plant growth/development, and tolerance against stresses in *S. aralocaspica* and the similar species.

## Data Availability Statement

The original contributions presented in the study are included in the article/Supplementary Material, further inquiries can be directed to the corresponding author.

## Author Contributions

HL, JC, and GC designed the experiments and methodology. JC and HL wrote the manuscript. JC, GC, LW, and TM conducted the experiments and collected the data. JC analyzed the data. All authors contributed critically to the manuscript and gave final approval for publication.

## Conflict of Interest

The authors declare that the research was conducted in the absence of any commercial or financial relationships that could be construed as a potential conflict of interest.

## Publisher’s Note

All claims expressed in this article are solely those of the authors and do not necessarily represent those of their affiliated organizations, or those of the publisher, the editors and the reviewers. Any product that may be evaluated in this article, or claim that may be made by its manufacturer, is not guaranteed or endorsed by the publisher.

## Footnotes

1
http://pfam.xfam.org/

2
http://hmmer.janelia.org/

3
http://smart.embl-heidelberg.de/

4
http://www.ebi.ac.uk/interpro/

5
https://phytozome.jgi.doe.gov

6
http://gsds.cbi.pku.edu.cn/

7
http://meme-suite.org/tools/meme

8
http://bioinformatics.psb.ugent.be/webtools/plantcare/html/

9
http://www.unafold.org/

10
http://web.expasy.org/compute_pi/

11
http://www.csbio.sjtu.edu.cn/bioinf/plant-multi/

12
https://abi-services.informatik.uni-tuebingen.de/yloc/webloc.cgi

13
https://www.ncbi.nlm.nih.gov/tools/primer-blast/

## Supplementary Material

Supplementary Figure 1 Constructs of plant expression vectors with a series of 5′-end deletion of *SaPEPC-1* and *SaPEPC-2* promoter sequences. **(A–D)** *SaPEPC-1*; **(E–H)** *SaPEPC-2*; **(A,E)** A schematic diagram of deletions at the 5′-end of two promoters; **(B,F)** A schematic diagram of promoter fragments fused with β-glucuronidase (GUS) gene; **(C,G)** PCR amplification of truncated promoter fragments; **(D,H)** Identification of the plant expression vector pBI121 replaced by different phosphoenolpyruvate carboxylase (PEPC) promoter fragments for CaMV35S promoter. Red box represents the light-response elements; light green box represents the stress-related elements; yellow box represents the endosperm-expressing elements; grass green box represents the root-specific expression and mesophyll cell development-related elements; purple box represents a high-level transcriptional element of 5′-UTR; the double italic slash represents the truncated site. M1, DL 5000 Marker; M2, DL 15000 Marker; M3, DL 15000 + 5000 Marker.

Supplementary Figure 2 Analysis of exons and introns in *PEPC* genes of different plant species. **(A)** Plant-type PEPC (PTPC) genes of dicots; **(B)** PTPC genes of monocots, mosses, and ferns; **(C)** Bacterial-type PEPC (BTPC) genes in plants.

Supplementary Figure 3 Distribution of conserved motifs in *PEPC* genes of different plant species. **(A)** PTPC genes of dicots; **(B)** PTPC genes of monocots, mosses, and ferns; **(C)** BTPC genes in plants; **(D)** Schematic diagram of base enrichment of top 10 motifs. The five-pointed star represents the active site of PEPC enzyme.

Supplementary Figure 4 Subcellular localization of SaPEPC-2 in tobacco epidermal cells. CBL1, calcineurin B-like protein 1, membrane marker control; ABI5, abscisic acid insensitive 5, nucleus marker control; GFP, green fluorescent protein. Bar = 20 μm.

Supplementary Figure 5 Analysis of 5′-UTR sequence of two *SaPEPC* promoters. **(A,B)** *SaPEPC-1*; **(C,D)** *SaPEPC-2.* **(A,C)** Secondary structure of DNA; **(B,D)** Secondary structure of RNA. The free energy (Δ*G*) of respective structure is shown.

Supplementary Figure 6 Ectopic expression of *SaPEPC-1* and *SaPEPC-2* in *Escherichia coli* and the immunoblotting analysis. **(A)** Sodium dodecyl sulfate–polyacrylamide gel electrophoresis (SDS-PAGE) analysis of recombinant SaPEPC proteins; **(B)** Immunoblotting analysis of the recombinant SaPEPC proteins with anti-His antibody. NI, non-induced total protein; Total, total crude protein; Sol, soluble protein; Ins, insoluble protein; lane M1, protein molecular weight (MW) marker; and lane M2, prestained protein MW marker. The arrowhead indicates the specific protein band of approximately 110 kDa corresponding to PEPC derivatives.

Supplementary Figure 7 Time courses of the growth of *SaPEPC-1* **(I)** and *SaPEPC-2* **(II)** recombinant strains under different abiotic stresses. **(A,A′)** Non-stressed condition; **(B,B′)** 400 mmol⋅L^–1^ NaCl; **(C,C′)** 10% PEG; **(D,D′)** 25°C (30°C for *SaPEPC-2*); **(E,E′)** 75 μmol⋅L^–1^ methyl viologen (MV); and **(F,F′)** pH 5.0. The culture was sampled at an interval of 2 h to a total of 12 h. Values are means ± SD of three replicate.

Supplementary Figure 8 Growth and enzyme activity of recombinant *SaPEPC* strains under temperature and MV treatments for 12 h. **(A)** Growth of recombinant *SaPEPC-1* and *SaPEPC-2* strains under different temperatures. **(B)** Enzyme activity of recombinant *SaPEPC-1* and *SaPEPC-2* strains under MV stress. *, ***, ****: Indicate a significant difference between control strain and recombinant *SaPEPC* strains at the same temperature at 0.05, 0.001, 0.0001 level, respectively. Different lowercase letters indicate significant differences between control and stress treatment at different MV concentrations. Values are means ± SD of three replicates.
