# Supplementary figures and images for: Genome-Wide Identification and Analysis of the Phosphoenolpyruvate Carboxylase Gene Family in Suaeda aralocaspica, an Annual Halophyte With Single-Cellular C4 Anatomy
Source: Front Plant Sci. 2021 Aug 30;12:665279. doi: 10.3389/fpls.2021.665279 (PMC8435749; doi:10.3389/fpls.2021.665279)

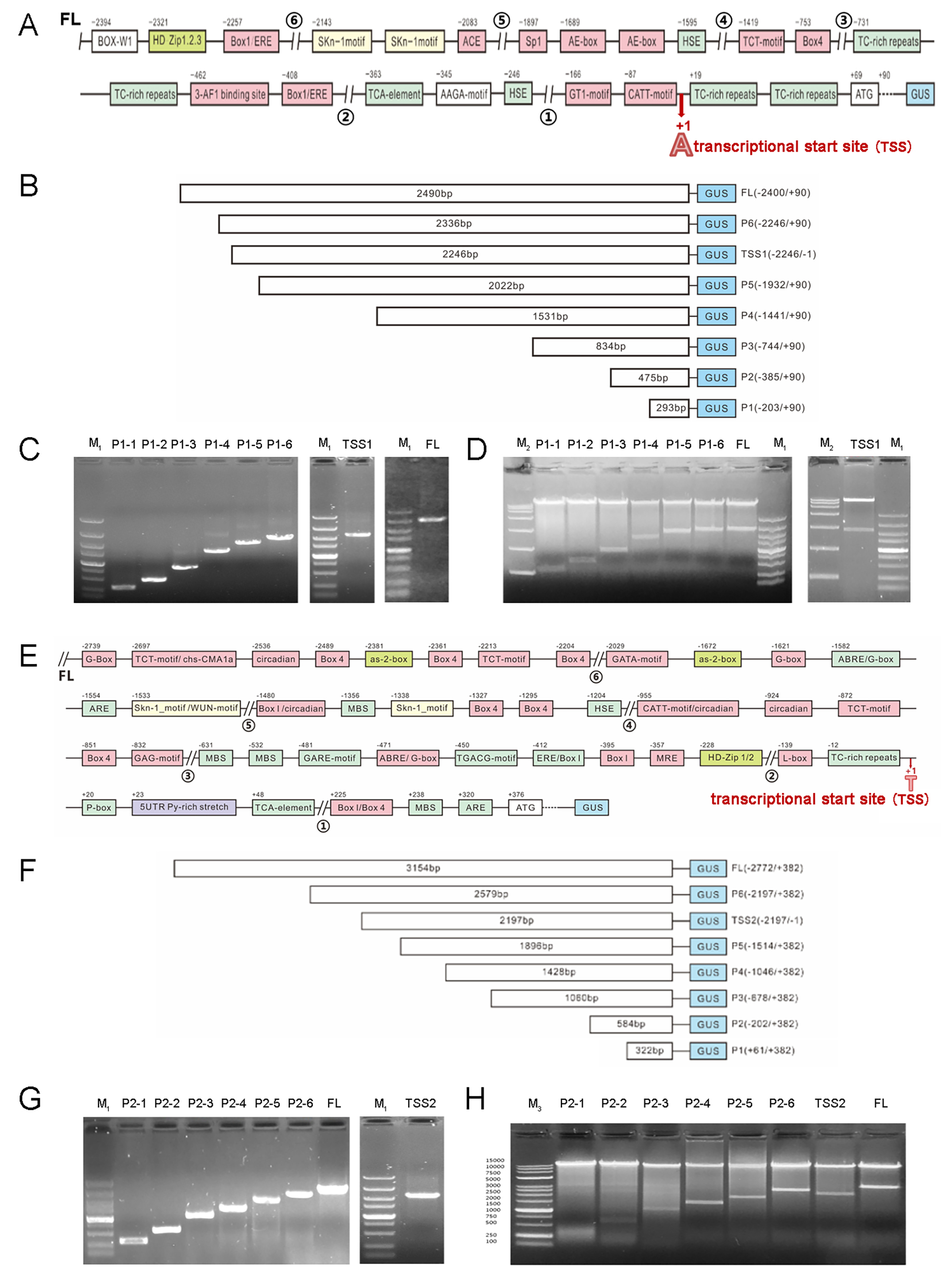

Supplement: Supplementary Figure 1 — Constructs of plant expression vectors with a series of 5′-end deletion of SaPEPC-1 and SaPEPC-2 promoter sequences. (A–D) SaPEPC-1; (E–H) SaPEPC-2; (A,E) A schematic diagram of deletions at the 5′-end of two promoters; (B,F) A schematic diagram of promoter fragments fused with β-glucuronidase (GUS) gene; (C,G) PCR amplification of truncated promoter fragments; (D,H) Identification of the plant expression vector pBI121 replaced by different phosphoenolpyruvate carboxylase (PEPC) promoter fragments for CaMV35S promoter. Red box represents the light-response elements; light green box represents the stress-related elements; yellow box represents the endosperm-expressing elements; grass green box represents the root-specific expression and mesophyll cell development-related elements; purple box represents a high-level transcriptional element of 5′-UTR; the double italic slash represents the truncated site. M1, DL 5000 Marker; M2, DL 15000 Marker; M3, DL 15000 + 5000 Marker. [file Image_1.JPEG]

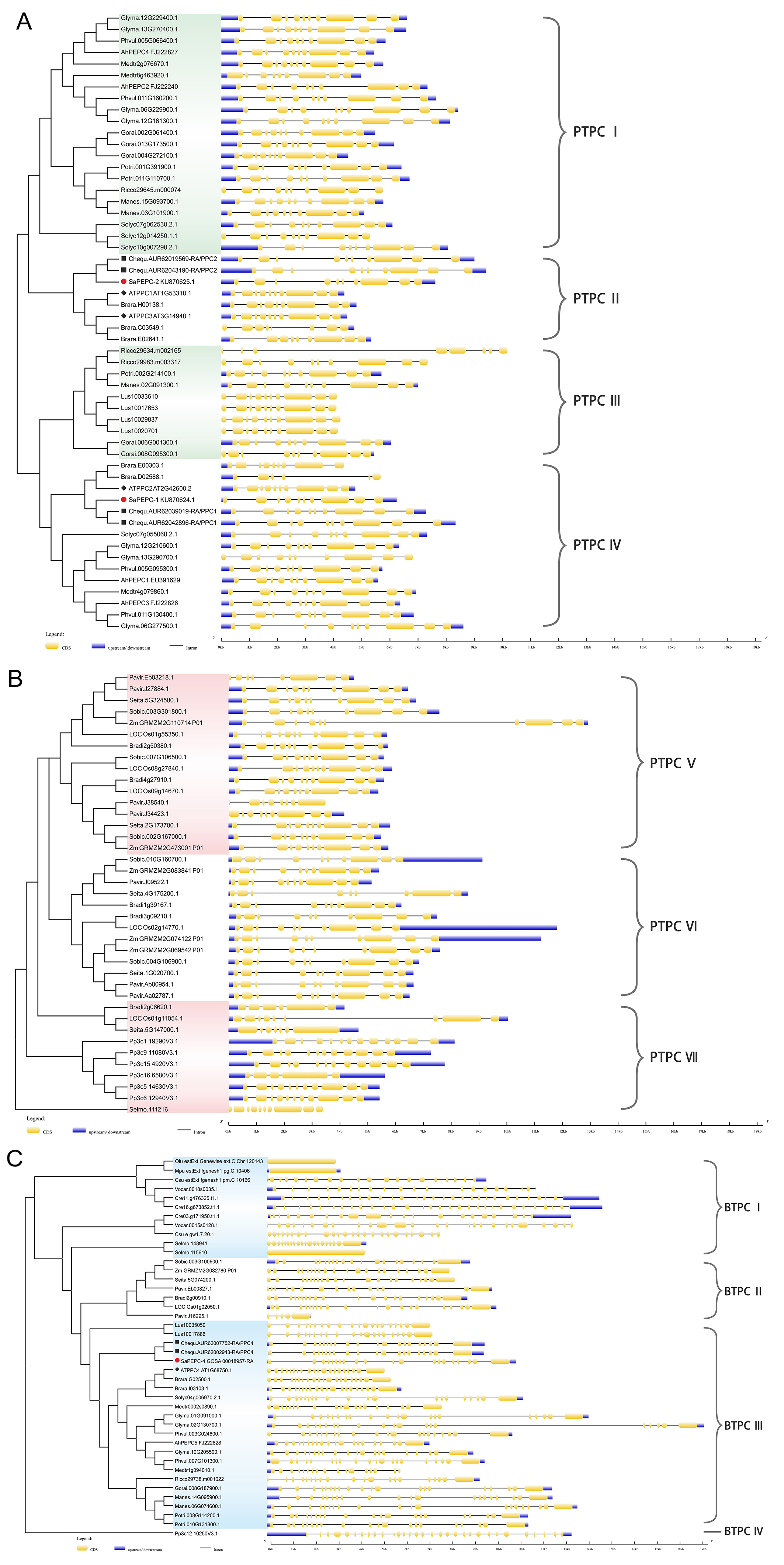

Supplement: Supplementary Figure 2 — Analysis of exons and introns in PEPC genes of different plant species. (A) Plant-type PEPC (PTPC) genes of dicots; (B) PTPC genes of monocots, mosses, and ferns; (C) Bacterial-type PEPC (BTPC) genes in plants. [file Image_2.jpg]

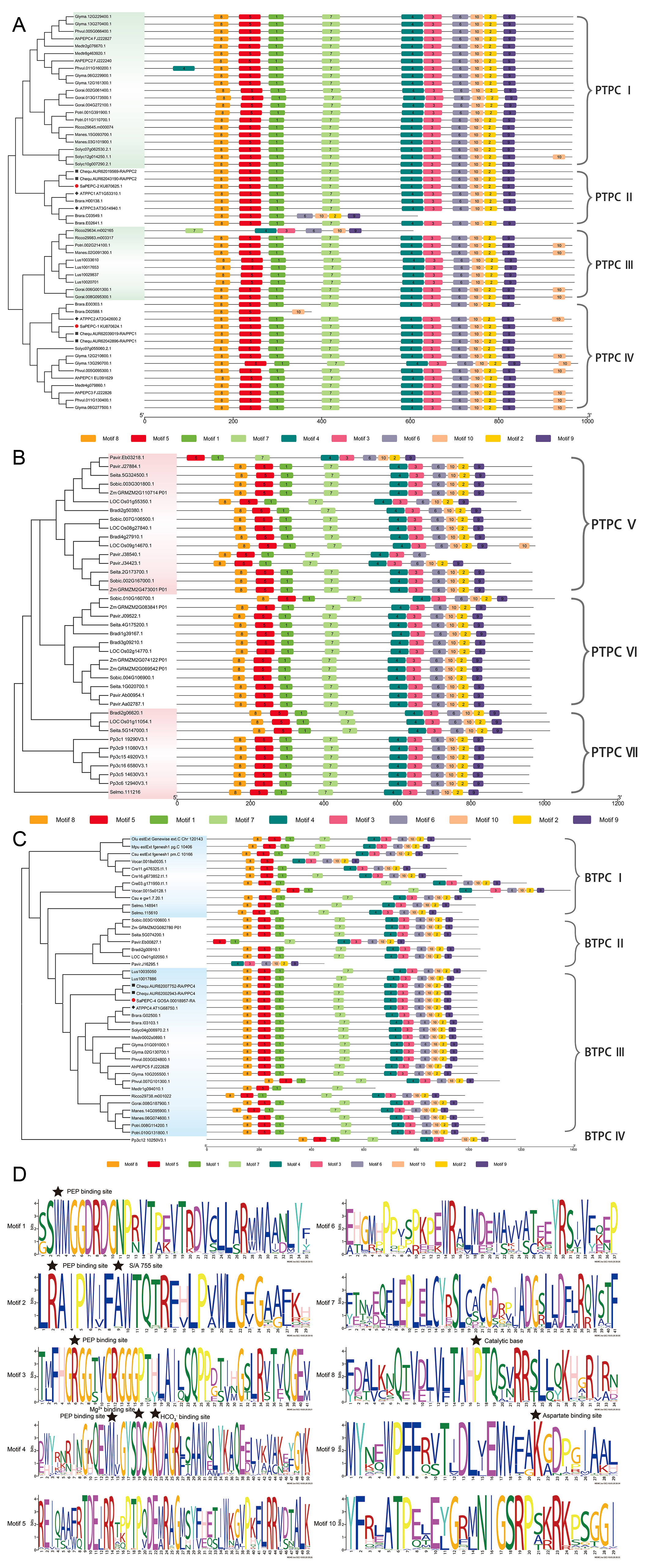

Supplement: Supplementary Figure 3 — Distribution of conserved motifs in PEPC genes of different plant species. (A) PTPC genes of dicots; (B) PTPC genes of monocots, mosses, and ferns; (C) BTPC genes in plants; (D) Schematic diagram of base enrichment of top 10 motifs. The five-pointed star represents the active site of PEPC enzyme. [file Image_3.jpg]

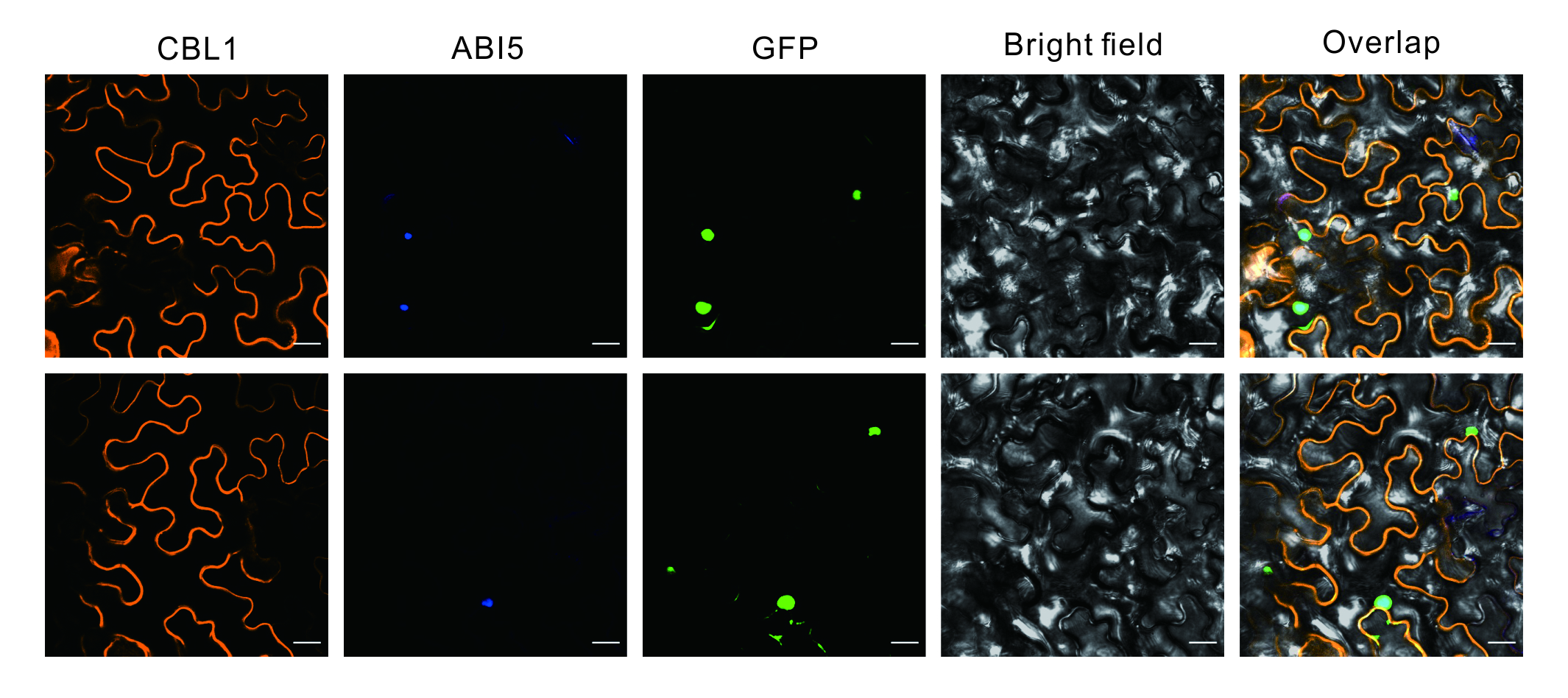

Supplement: Supplementary Figure 4 — Subcellular localization of SaPEPC-2 in tobacco epidermal cells. CBL1, calcineurin B-like protein 1, membrane marker control; ABI5, abscisic acid insensitive 5, nucleus marker control; GFP, green fluorescent protein. Bar = 20 μm. [file Image_4.jpg]

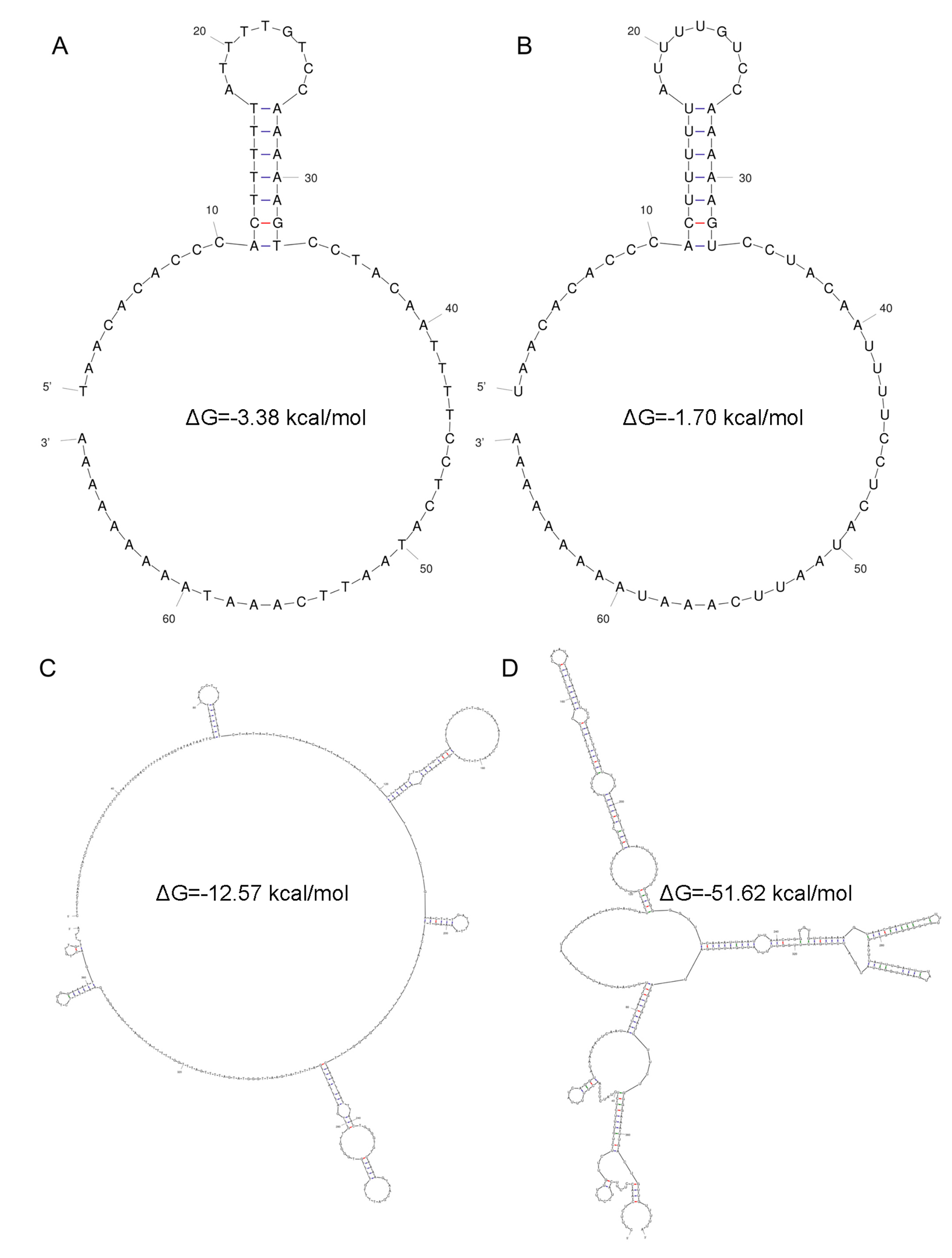

Supplement: Supplementary Figure 5 — Analysis of 5′-UTR sequence of two SaPEPC promoters. (A,B) SaPEPC-1; (C,D) SaPEPC-2. (A,C) Secondary structure of DNA; (B,D) Secondary structure of RNA. The free energy (ΔG) of respective structure is shown. [file Image_5.jpg]

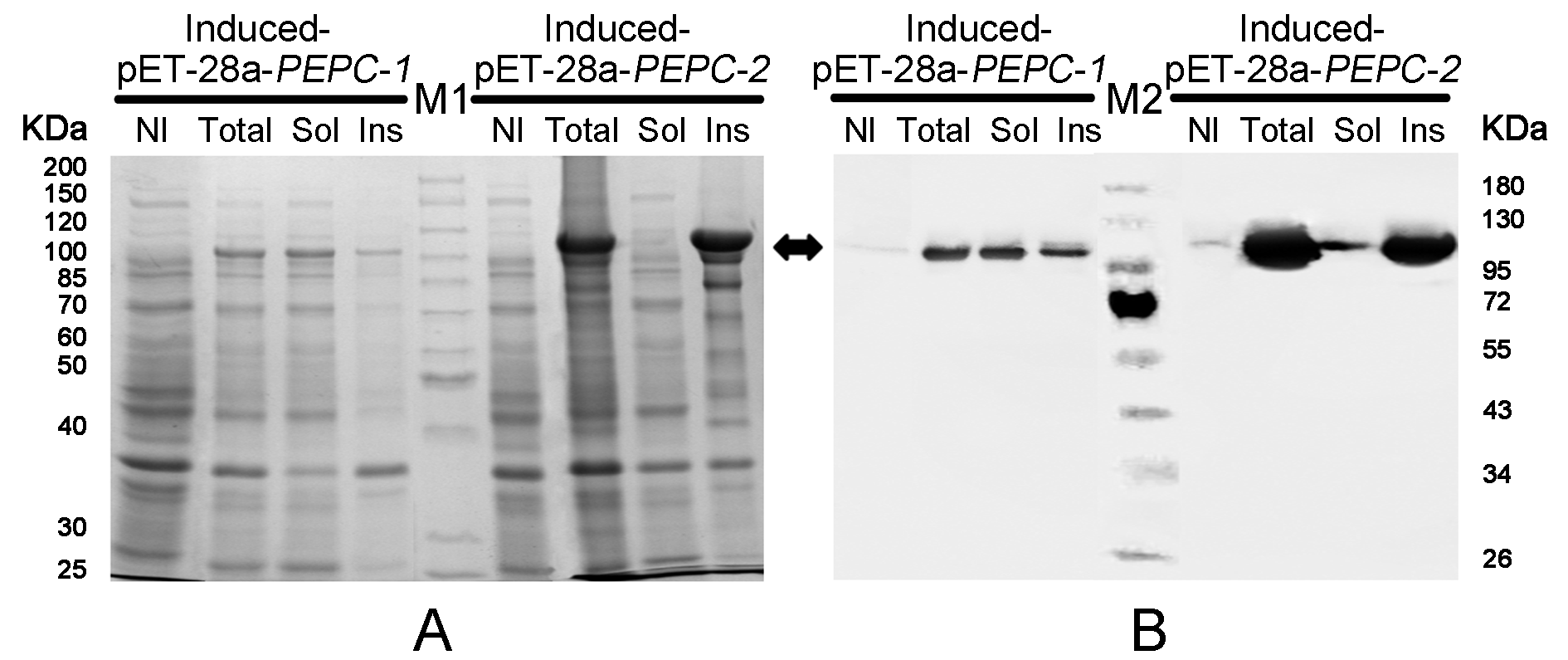

Supplement: Supplementary Figure 6 — Ectopic expression of SaPEPC-1 and SaPEPC-2 in Escherichia coli and the immunoblotting analysis. (A) Sodium dodecyl sulfate–polyacrylamide gel electrophoresis (SDS-PAGE) analysis of recombinant SaPEPC proteins; (B) Immunoblotting analysis of the recombinant SaPEPC proteins with anti-His antibody. NI, non-induced total protein; Total, total crude protein; Sol, soluble protein; Ins, insoluble protein; lane M1, protein molecular weight (MW) marker; and lane M2, prestained protein MW marker. The arrowhead indicates the specific protein band of approximately 110 kDa corresponding to PEPC derivatives. [file Image_6.jpg]

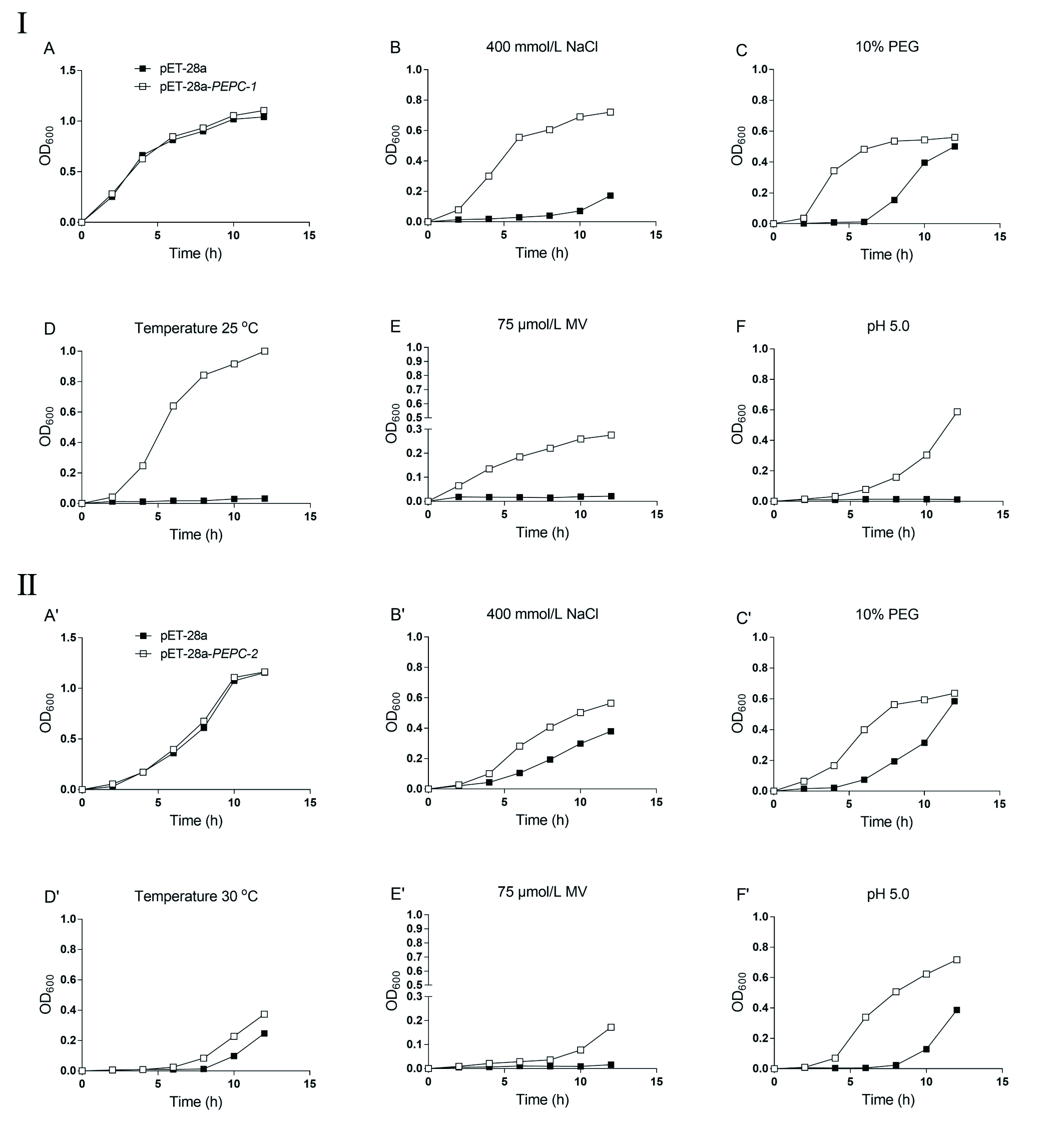

Supplement: Supplementary Figure 7 — Time courses of the growth of SaPEPC-1 (I) and SaPEPC-2 (II) recombinant strains under different abiotic stresses. (A,A′) Non-stressed condition; (B,B′) 400 mmol⋅L–1 NaCl; (C,C′) 10% PEG; (D,D′) 25°C (30°C for SaPEPC-2); (E,E′) 75 μmol⋅L–1 methyl viologen (MV); and (F,F′) pH 5.0. The culture was sampled at an interval of 2 h to a total of 12 h. Values are means ± SD of three replicate. [file Image_7.jpg]

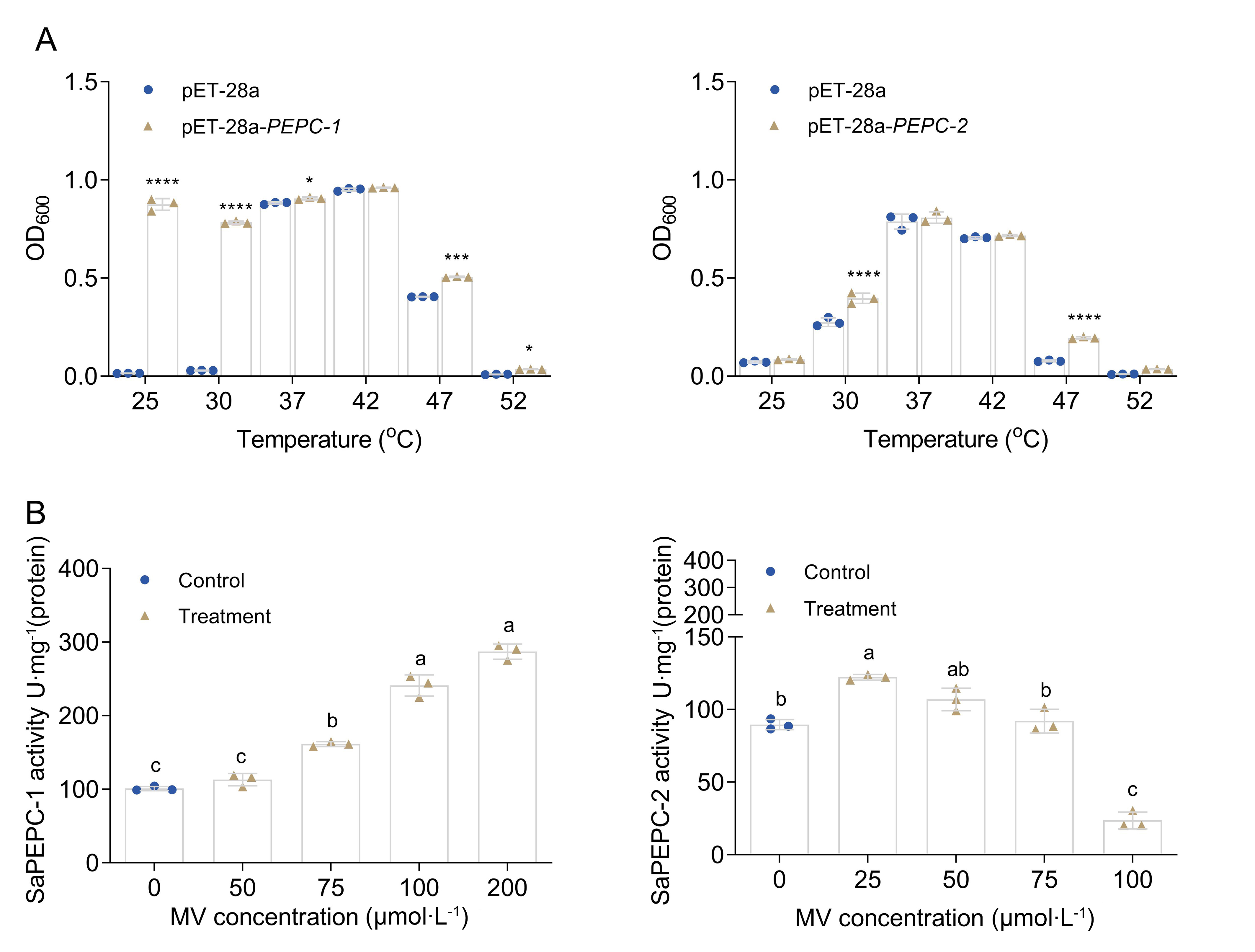

Supplement: Supplementary Figure 8 — Growth and enzyme activity of recombinant SaPEPC strains under temperature and MV treatments for 12 h. (A) Growth of recombinant SaPEPC-1 and SaPEPC-2 strains under different temperatures. (B) Enzyme activity of recombinant SaPEPC-1 and SaPEPC-2 strains under MV stress. *, ***, ****: Indicate a significant difference between control strain and recombinant SaPEPC strains at the same temperature at 0.05, 0.001, 0.0001 level, respectively. Different lowercase letters indicate significant differences between control and stress treatment at different MV concentrations. Values are means ± SD of three replicates. [file Image_8.jpeg]
