# Supplementary material for: Genome-Wide Identification and Analysis of the Phosphoenolpyruvate Carboxylase Gene Family in Suaeda aralocaspica, an Annual Halophyte With Single-Cellular C4 Anatomy
Source: Front Plant Sci. 2021 Aug 30;12:665279. doi: 10.3389/fpls.2021.665279 (PMC8435749; doi:10.3389/fpls.2021.665279)
Supplement: Supplementary file 9 [file Table_1.docx]

**Supplementary Table 1 *In vivo* and *in vitro* experimental design**

| Experiment | Treatment | | Sampling time and tissue |
| --- | --- | --- | --- |
| ***In vivo*** | | | |
| qRT-PCR  (Germination) | Developmental stages | 0 h, 8 h, 12 h, 24 h, 2 d, 5 d, 10 d, 15 d  Light vs Dark | Dry seeds, germinated for 8 h, 12 h, 24 h, 2 d, 5 d, 10 d, 15 d (Germinated seeds)  Seedlings germinated for 7 d (Cotyledon, hypocotyl, radicle) |
|  | Stress treatments | 0, 100, 300, 500 mmol·L^-1^ NaCl  0, 200, 600, 1000 mmol·L^-1^ Mannitol  0, 1, 5, 10 μmol·L^-1^ ABA  Light vs Dark | Seedlings germinated for 7 d (Whole seedlings) |
|  | Light intensities | 30, 300, 900 µmol·m^-2^·s^-1^ | Seedlings germinated for 7 d (Whole seedlings) |
| qRT-PCR  (Seedling) | Developmental stages | 3 d, 15 d, 30 d, 60 d | 3 d, 15 d, 30 d, 60 d after emergence  (Whole seedlings) |
|  |  |  | 15 d and 90 d after emergence  (Leaf, stem, root of seedlings/adult plants) |
| PEPC activity | Developmental stages | 3 d, 15 d, 30 d, 60 d | 3 d, 15 d, 30 d, 60 d after emergence  (Cotyledons/leaves) |
|  | Stress treatments | 0, 100, 300, 500 mmol·L^-1^ NaCl | 15 d after emergence (Cotyledons/leaves) |
|  |  | 7 d, 14 d, 28 d natural drought | 30 d seedlings naturally dried for 7 d, 14 d, 28d (Leaves) |
|  | Photoperiods | 8:00 to 22:00 within the same day | 60 d plants cultivated for 2-3 d in continual sunny days (Leaves) |
| Photosynthetic enzymes accumulation | Photoperiods | 8:00 to 22:00 within the same day | 60 d plants cultivated for 2-3 d in continual sunny days (Leaves) |
| ***In vitro*** | | | |
| Subcellular localization | pCAMBIA1300*-35S*::*SaPEPC-1-eGFP*  pCAMBIA1300*-35S*::*SaPEPC-2-eGFP* | | Five- to six-week-old *Nicotiana benthamiana* leaves were prepared for infiltration. Treated plants were held in the dark overnight, and then transferred to normal growth conditions for another 48 h (Leaves) |
| Promoter activity | A series of 5’- deletion of *SaPEPC-1* promoters:  P1-1/2//3/4/5/6/FL; TSS1  A series of 5’- deletion of *SaPEPC-2* promoters:  P2-1/2//3/4/5/6/FL; TSS2  Light vs Dark | | Five- to six-week-old *N. benthamiana* leaves were prepared for infiltration. Treated tobacco were exposed to normal illumination and darkness for 3 d in growth chamber (Leaves) |
| Recombinant protein activity | Stress treatments | 400 mmol·L^-1^ NaCl, 10% (w/v) PEG, 25 µmol·L^-1^ methyl viologen (oxidative stress), 30°C, pH 5.0 | An interval of 3 h to a total of 12 h to measure enzyme activity  (*E. coli* cultures) |
|  | Substrates | 0.5, 1, 3, 4, 5 mmol·L^-1^ PEP  0.5, 3, 5, 10, 20 mmol·L^-1^ NaHCO_3_  0.5, 3, 5, 10, 20 mmol·L^-1^ MgCl_2_ | Harvested after 12 h growth  (*E. coli* cultures) |
|  | pH | pH 7.0, 7.3, 8.0, 8.3, 9.0, 10.0 |  |
|  | Temperatures | 15, 20, 25, 30, 35, 40, 45, 50, 55°C |  |
|  | Metal ion | 10 mmol·L^-1^ MgCl_2_, CuCl_2_, AlCl_3_, MnSO_4_, EDTA |  |
|  | Metabolic effectors | 0, 5, 10, 20, 40 mmol·L^-1^ glucose-6-phosphate  0, 5, 10, 20, 40 mmol·L^-1^ glycine  0, 2, 5, 10, 15, 20, 40 mmol·L^-1^ malate |  |

**Supplementary Table 2 *PEPC* gene information of 26 representative species used to construct the phylogenetic tree**

| Species | Gene ID | Species | Gene ID |
| --- | --- | --- | --- |
| *Arabidopsis thaliana* | ATPPC1 AT1G53310.1 | *Panicum virgatum* | Pavir.J38540.1 |
|  | ATPPC2 AT2G42600.2 |  | Pavir.J34423.1 |
|  | ATPPC3 AT3G14940.1 |  | Pavir.Eb03218.1 |
|  | ATPPC4 AT1G68750.1 |  | Pavir.J27884.1 |
| *Arachis hypogaea* | AhPEPC1 EU391629 |  | Pavir.J09522.1 |
|  | AhPEPC2 FJ222240 |  | Pavir.Ab00954.1 |
|  | AhPEPC3 FJ222826 |  | Pavir.Aa02787.1 |
|  | AhPEPC4 FJ222827 |  | Pavir.Eb00827.1 |
|  | AhPEPC5 FJ222828 |  | Pavir.J16295.1 |
| *Brachypodium distachyon* | Bradi4g27910.1 | *Phaseolus vulgaris* | Phvul.011G160200.1 |
|  | Bradi2g50380.1 |  | Phvul.005G066400.1 |
|  | Bradi1g39167.1 |  | Phvul.005G095300.1 |
|  | Bradi2g06620.1 |  | Phvul.011G130400.1 |
|  | Bradi3g09210.1 |  | Phvul.003G024800.1 |
|  | Bradi2g00910.1 |  | Phvul.007G101300.1 |
| *Brassica rapa* | Brara.H00138.1 | *Physcomitrella patens* | Pp3c1_19290V3.1 |
|  | Brara.C03549.1 |  | Pp3c15_4920V3.1 |
|  | Brara.E02641.1 |  | Pp3c9_11080V3.1 |
|  | Brara.E00303.1 |  | Pp3c16_6580V3.1 |
|  | Brara.D02588.1 |  | Pp3c5_14630V3.1 |
|  | Brara.G02500.1 |  | Pp3c6_12940V3.1 |
|  | Brara.I03103.1 |  | Pp3c12_10250V3.1 |
| *Chenopodium quinoa* | Chequ.AUR62039019 | *Populus trichocarpa* | Potri.001G391900.1 |
|  | Chequ.AUR62042896 |  | Potri.011G110700.1 |
|  | Chequ.AUR62019569 |  | Potri.002G214100.1 |
|  | Chequ.AUR62043190 |  | Potri.008G114200.1 |
|  | Chequ.AUR62007752 |  | Potri.010G131800.1 |
|  | Chequ.AUR62002943 | *Ricinus communis* | Ricco_29634.m002165 |
| *Glycine max* | Glyma.06G229900.1 |  | Ricco_29645.m000074 |
|  | Glyma.12G161300.1 |  | Ricco_29983.m003317 |
|  | Glyma.12G229400.1 |  | Ricco_29738.m001022 |
|  | Glyma.13G270400.1 | *Setaria italica* | Seita.2G173700.1 |
|  | Glyma.12G210600.1 |  | Seita.5G324500.1 |
|  | Glyma.13G290700.1 |  | Seita.4G175200.1 |
|  | Glyma.06G277500.1 |  | Seita.1G020700.1 |
|  | Glyma.10G205500.1 |  | Seita.5G147000.1 |
|  | Glyma.01G091000.1 |  | Seita.5G074200.1 |
|  | Glyma.02G130700.1 | *Solanum lycopersicum* | Solyc12g014250.1.1 |
| *Gossypium raimondii* | Gorai.002G061400.1 |  | Solyc07g055060.2.1 |
|  | Gorai.013G173500.1 |  | Solyc07g062530.2.1 |
|  | Gorai.004G272100.1 |  | Solyc10g007290.2.1 |
|  | Gorai.006G001300.1 |  | Solyc04g006970.2.1 |
|  | Gorai.008G095300.1 | *Sorghum bicolor* | Sobic.007G106500.1 |
|  | Gorai.008G187900.1 |  | Sobic.002G167000.1 |
| *Linum usitatissimum* | Lus10033610.g |  | Sobic.003G301800.1 |
|  | Lus10017653.g |  | Sobic.010G160700.1 |
|  | Lus10029837.g |  | Sobic.004G106900.1 |
|  | Lus10020701.g |  | Sobic.003G100600.1 |
|  | Lus10035050.g | *Volvox carteri* | Vocar.0018s0035.1 |
|  | Lus10017886.g |  | Vocar.0015s0128.1 |
| *Manihot esculenta* | Manes.15G093700.1 | *Zea mays* | GRMZM2G069542_P01 |
|  | Manes.03G101900.1 |  | (ZmPEPC1) |
|  | Manes.02G091300.1 |  | GRMZM2G074122_P01 |
|  | Manes.06G074600.1 |  | (ZmPEPC2) |
|  | Manes.14G095900.1 |  | GRMZM2G082780_P01 |
| *Medicago truncatula* | Medtr8g463920.1 |  | (ZmPEPC3) |
|  | Medtr2g076670.1 |  | GRMZM2G083841_P01 |
|  | Medtr4g079860.1 |  | (ZmPEPC4) |
|  | Medtr1g094010.1 |  | GRMZM2G110714_P01 |
|  | Medtr0002s0890.1 |  | (ZmPEPC5) |
| *Oryza sativa* | LOC_Os08g27840.1 |  | GRMZM2G473001_P01 |
|  | LOC_Os09g14670.1 |  | (ZmPEPC6) |
|  | LOC_Os01g55350.1 |  |  |
|  | LOC_Os02g14770.1 |  |  |
|  | LOC_Os01g11054.1 |  |  |
|  | LOC_Os01g02050.1 |  |  |
| *Coccomyxa subellipsoidea* | Csu_estExt_fgenesh1_pm.C_10166 |  |  |
|  | Csu_e_gw1.7.20.1 |  |  |
| *Chlamydomonas reinhardtii* | Cre16.g673852.t1.1 |  |  |
|  | Cre11.g476325.t1.1 |  |  |
|  | Cre03.g171950.t1.1 |  |  |
| *Ostreococcus lucimarinus* | Olu_estExt_Genewise_ext.C_Chr_120143 |  |  |
| *Micromonas pusilla* | Mpu_estExt_fgenesh1_pg.C_10406 |  |  |
| *Selaginella moellendorffii* | Selmo_111216 |  |  |
|  | Selmo_115610 |  |  |
|  | Selmo_148941 |  |  |

**Supplementary Table 3 Primer sequences used in the present study**

| Primer name | Primer sequence （5’→3’） | Fragment (bp) | |
| --- | --- | --- | --- |
| Super1300-*PEPC*1-F | GCGTCGACATGGCAACTGTTAAGTTGGAGAGACT | | 2901 |
| Super1300-*PEPC*s-R | CGGGGTACCACCGGTGTTCTGCATTC | |  |
| Super1300-*PEPC*2-F | GCGTCGACATGGCGCGGAATATTGAGAAAATGG | | 2901 |
| Ppc1-F1 | CCCAAGCTTTTCCCCACATCAAAAAACAAGTTGCTC | | 293 |
| Ppc1-F2 | CCCAAGCTTCTAGACTTTGATTAAGGAAAGAGTGAAG | | 475 |
| Ppc1-F3 | CCCAAGCTTCATCACTACTAGAATTTTCTTTATACTTTC | | 834 |
| Ppc1-F4 | CCCAAGCTTGATCTCATGCGATTATGTTTTGTCTTAC | | 1531 |
| Ppc1-F5 | CCCAAGCTTCCAACGATTAAAGATTATGATTGCTATG | | 2022 |
| Ppc1-F6 | CCCAAGCTTCGTAGTACTTCCCCAATCTTAATTTAG | | 2336 |
| Ppc1-FLF | CCCAAGCTTCATAATTTGACCAATAGCATAAAAAACTG | | 2490 |
| Ppc1-FLR | CGCGGATCCCTCCAACTTAACAGTTGCCAA | |  |
| Ppc1-R_TSS_ | CGCGGATCCGAATATATATAGGGTTGATTTTTGG | | 2246 |
| Ppc2-F1 | CCCAAGCTTGGTATAATAATTCAATAATTCAACCTTT | | 322 |
| Ppc2-F2 | CCCAAGCTTGGCATCGTTGAAAAGCATATTCTCAC | | 584 |
| Ppc2-F3 | CCCAAGCTTCCGGAATCTCAATTTTCCAATACAG | | 1060 |
| Ppc2-F4 | CCCAAGCTTGCCACAAGGGCAGATATGAGAATTG | | 1428 |
| Ppc2-F5 | CCCAAGCTTGTCCCAAATTCCCAATGTAGAACC | | 1896 |
| Ppc2-F6 | CCCAAGCTTGATTTTCTGTGTACGAGTGCCGTAAG | | 2579 |
| Ppc2-FLF | CCCAAGCTTGATGGCATGTTGCGTTAGTAGAATG | | 3154 |
| Ppc2-FLR | CGCGGATCCCGCCATTAAAAACAACAGTC | |  |
| Ppc2-R_TSS_ | CGCGGATCCAAGTTAGAGAAGTAGAGTGAC | | 2197 |
| *SaPPC1*-qRT-F | GCATCCACCAAGCTCTCCTAAG | | 147 |
| *SaPPC1*-qRT-R | CCATACTCCAACTCAGGCG | |  |
| *SaPPC2*-qRT-F | GAACTCTCCAGCGTTTCACTG | | 188 |
| *SaPPC2*-qRT-R | CAATGCAGTTTCTGGTGTGG | |  |
| *β-tubulin*-qRT-F | CCTTATTCCATTCCCCAGGCTTC | | 223 |
| *β-tubulin*-qRT-R | CATCTGCTCATCAACCTCCTTTGTGC | |  |
| pET28a-*PEPC1*-F | CCGGAATTCATGGCAACTGTTAAGTTGG | | 2901 |
| pET28a-*PEPCs*-R | TTTGCGGCCGCACCGGTGTTCTGCATTC | |  |
| pET28a-*PEPC2*-F | CGTCGACTTATGGCGCGGAATATTGAGAAA | | 2901 |

**Supplementary Table 4 Protein distribution of *PEPC* gene family in different plant species**

| Species | PTPCⅠ | PTPCⅡ | PTPCⅢ | PTPCⅣ | PTPCⅤ | PTPCⅥ | PTPCⅦ | BTPCⅠ | BTPCⅡ | BTPCⅢ | BTPCⅣ | Total |
| --- | --- | --- | --- | --- | --- | --- | --- | --- | --- | --- | --- | --- |
| *Arabidopsis thaliana* | 0 | 2 | 0 | 1 | 0 | 0 | 0 | 0 | 0 | 1 | 0 | 4 |
| *Arachis hypogaea* | 2 | 0 | 0 | 2 | 0 | 0 | 0 | 0 | 0 | 1 | 0 | 5 |
| *Brachypodium distachyon* | 0 | 0 | 0 | 0 | 2 | 2 | 1 | 0 | 1 | 0 | 0 | 6 |
| *Brassica rapa* | 0 | 3 | 0 | 2 | 0 | 0 | 0 | 0 | 0 | 2 | 0 | 7 |
| *Chenopodium quinoa* | 0 | 2 | 0 | 2 | 0 | 0 | 0 | 0 | 0 | 2 | 0 | 6 |
| *Chlamydomonas reinhardtii* | 0 | 0 | 0 | 0 | 0 | 0 | 0 | 3 | 0 | 0 | 0 | 3 |
| *Coccomyxa subellipsoidea* | 0 | 0 | 0 | 0 | 0 | 0 | 0 | 2 | 0 | 0 | 0 | 2 |
| *Glycine max* | 4 | 0 | 0 | 3 | 0 | 0 | 0 | 0 | 0 | 3 | 0 | 10 |
| *Gossypium raimondii* | 3 | 0 | 2 | 0 | 0 | 0 | 0 | 0 | 0 | 1 | 0 | 6 |
| *Linum usitatissimum* | 0 | 0 | 4 | 0 | 0 | 0 | 0 | 0 | 0 | 2 | 0 | 6 |
| *Manihot esculenta* | 2 | 0 | 1 | 0 | 0 | 0 | 0 | 0 | 0 | 2 | 0 | 5 |
| *Medicago truncatula* | 2 | 0 | 0 | 1 | 0 | 0 | 0 | 0 | 0 | 2 | 0 | 5 |
| *Micromonas pusilla* | 0 | 0 | 0 | 0 | 0 | 0 | 0 | 1 | 0 | 0 | 0 | 1 |
| *Oryza sativa* | 0 | 0 | 0 | 0 | 3 | 1 | 1 | 0 | 1 | 0 | 0 | 6 |
| *Ostreococcus lucimarinus* | 0 | 0 | 0 | 0 | 0 | 0 | 0 | 1 | 0 | 0 | 0 | 1 |
| *Panicum virgatum* | 0 | 0 | 0 | 0 | 4 | 3 | 0 | 0 | 2 | 0 | 0 | 9 |
| *Phaseolus vulgaris* | 2 | 0 | 0 | 2 | 0 | 0 | 0 | 0 | 0 | 2 | 0 | 6 |
| *Physcomitrella patens* | 0 | 0 | 0 | 0 | 0 | 0 | 6 | 0 | 0 | 0 | 1 | 7 |
| *Populus trichocarpa* | 2 | 0 | 1 | 0 | 0 | 0 | 0 | 0 | 0 | 2 | 0 | 5 |
| *Ricinus communis* | 1 | 0 | 2 | 0 | 0 | 0 | 0 | 0 | 0 | 1 | 0 | 4 |
| *Selaginella moellendorffii* | 0 | 0 | 0 | 0 | 0 | 0 | 1 | 2 | 0 | 0 | 0 | 3 |
| *Setaria italica* | 0 | 0 | 0 | 0 | 2 | 2 | 1 | 0 | 1 | 0 | 0 | 6 |
| *Solanum lycopersicum* | 3 | 0 | 0 | 1 | 0 | 0 | 0 | 0 | 0 | 1 | 0 | 5 |
| *Sorghum bicolor* | 0 | 0 | 0 | 0 | 3 | 2 | 0 | 0 | 1 | 0 | 0 | 6 |
| ***Suaeda aralocaspica*** | 0 | 1 | 0 | 1 | 0 | 0 | 0 | 0 | 0 | 1 | 0 | 3 |
| *Volvox carteri* | 0 | 0 | 0 | 0 | 0 | 0 | 0 | 2 | 0 | 0 | 0 | 2 |
| *Zea mays* | 0 | 0 | 0 | 0 | 2 | 3 | 0 | 0 | 1 | 0 | 0 | 6 |
| Total no. | 21 | 8 | 10 | 15 | 16 | 13 | 10 | 11 | 7 | 23 | 1 | 135 |

**Supplementary Table 5 The top ten conserved motifs of 135 PEPC proteins** **from 27 different plant species**

| Motif | AA | Sequence |
| --- | --- | --- |
| Motif-1 | 35 | SS**W**MGGDRDGNPRVTPEVTRDVCLLARMMAANLYF |
| Motif-2 | 29 | L**R**AIPWIF**A**WTQTRFHLPVWLGFGAAFKH |
| Motif-3 | 41 | TMFHGRGGTVG**R**GGGPTHLAILSQPPDTIHGSLRVTVQGEV |
| Motif-4 | 50 | DWYRNRINGKQEV**M**IGYS**D**SG**K**DAGRLSAAWQLYKAQEELVKVAKEYGVK |
| Motif-5 | 50 | REIQAAFRTDEIRRTPPTPQDEMRAGMSYFHETIWKGVPKFLRRVDTALK |
| Motif-6 | 37 | EHGMHPPVSPKPEWRALMDEMAVVATEEYRSIVFQEP |
| Motif-7 | 41 | FTNVEQFLEPLELCYRSLCACGDRPIADGSLLDFLRQVSTF |
| Motif-8 | 34 | FDALKNQTVDLVLTA**H**PTQSVRRSLLQKHGRIRN |
| Motif-9 | 29 | MYNZWPFFRVTIDLVEMVFA**K**GDPGIAAL |
| Motif-10 | 29 | YFRLATPELEYGRMNIGSRPSKRKPSGGI |

Note: the underlines indicate the key domains of the catalytic reaction and binding sites for the substrate and inhibitor.

**Supplementary Table 6 Plant species lacking any of the top ten conserved motifs of the PEPC amino acid sequence**

| Species | Accession No. | Lacking motif | PEPC type |
| --- | --- | --- | --- |
| *Brassica rapa* | Brara.C03549.1 | 3, 4, 7 | PTPC Ⅱ |
|  | Brara.D02588.1 | 1-4, 6-7, 9 | PTPC Ⅳ |
| *Ricinus communis* | Ricco_29634.m002165 | 1-2, 5, 8 | PTPC Ⅲ |
| *Panicum virgatum* | Pavir.Eb03218.1 | 8 | PTPC Ⅴ |
|  | Pavir.J38540.1 | 2, 9-10 | PTPC Ⅴ |
|  | Pavir.Eb00827.1 | 8 | BTPC Ⅱ |
|  | Pavir.J16295.1 | 1, 5, 7-8 | BTPC Ⅱ |
| *Chlamydomonas reinhardtii* | Cre11.g476325.t1.1 | 7 | BTPC Ⅰ |
| *Volvox carteri* | Vocar.0018s0035.1 | 1, 7 | BTPC Ⅰ |
| *Medicago truncatula* | Medtr1g094010.1 | 2-4, 6, 9-10 | BTPC Ⅲ |
| *Physcomitrella patens* | Pp3c12_10250V3.1 | 6 | BTPC Ⅳ |

**Supplementary Table 7 *Cis*-elements predicted in *SaPEPC* gene promoter sequences by PlantCARE database**

| Motifs | Sequences | *SaPEPC-1* | *SaPEPC-2* | *SaPEPC-4* |
| --- | --- | --- | --- | --- |
| ***Light response related*** | | | | |
| AE-box | AGAAACAA | 2 |  |  |
| AT1-motif | AATTATTTTTTATT | 1 |  |  |
| ATCT-motif | AATCTAATCC |  |  | 1 |
| Box-4 | ATTAAT | 5 | 8 | 7 |
| chs-CMA1a | TTACTTAA |  | 1 |  |
| G-box | TACGTG |  | 2 | 1 |
| GA-motif | ATAGATAA |  |  | 1 |
| GT1-motif | GGTTAA | 3 |  |  |
| I-box | atGATAAGGTC | 1 |  |  |
| MRE | AACCTAA |  | 1 |  |
| TCCC-motif | TCTCCCT |  |  | 1 |
| TCT-motif | TCTTAC | 2 | 1 | 1 |
| ***Biotic and abiotic stress response related*** | | | | |
| STRE | AGGGG | 1 | 1 | 4 |
| TC-rich repeats | ATTCTCTAAC |  | 1 |  |
| WUN-motif | AAATTACT | 1 |  | 2 |
| ARE | AAACCA | 1 | 2 | 3 |
| LTR | CCGAAA |  | 1 |  |
| MBS | CAACTG | 1 | 3 | 1 |
| ***Morphology development and specific expression related*** | | | | |
| HD-Zip 1 | CAAT(A/T)ATTG | 1 | 1 | 1 |
| HD-Zip 3 | GTAAT(G/C)ATTAC | 1 |  |  |
| CAT-box | GCCACT | 2 |  | 1 |
| ***Phytohormone response related*** | | | | |
| ABRE | ACGTG |  | 2 | 3 |
| ABRE3a | TACGTG |  | 2 | 1 |
| ABRE4 | CACGTA |  | 2 | 1 |
| ERE | ATTTTAAA | 3 | 4 | 2 |
| TATC-box | TATCCCA |  | 1 |  |
| P-box | CCTTTTG |  | 1 | 1 |
| TGACG-motif | TGACG |  | 1 |  |
| CGTCA-motif | CGTCA |  | 1 |  |
| TCA-element | CCATCTTTTT |  | 1 | 4 |
| **Total number** | 30 | 25 | 37 | 36 |
