# Supplementary material for: Genome-Wide Identification and Analysis of the Phosphoenolpyruvate Carboxylase Gene Family in Suaeda aralocaspica, an Annual Halophyte With Single-Cellular C4 Anatomy
Source: Front Plant Sci. 2021 Aug 30;12:665279. doi: 10.3389/fpls.2021.665279 (PMC8435749; doi:10.3389/fpls.2021.665279)

1. **qRT-PCR Primer specificity (BLAST, DNA gel, melt-curve)**

(i) Primer-BLAST results of *SaPEPC-1* and *SaPEPC-2*


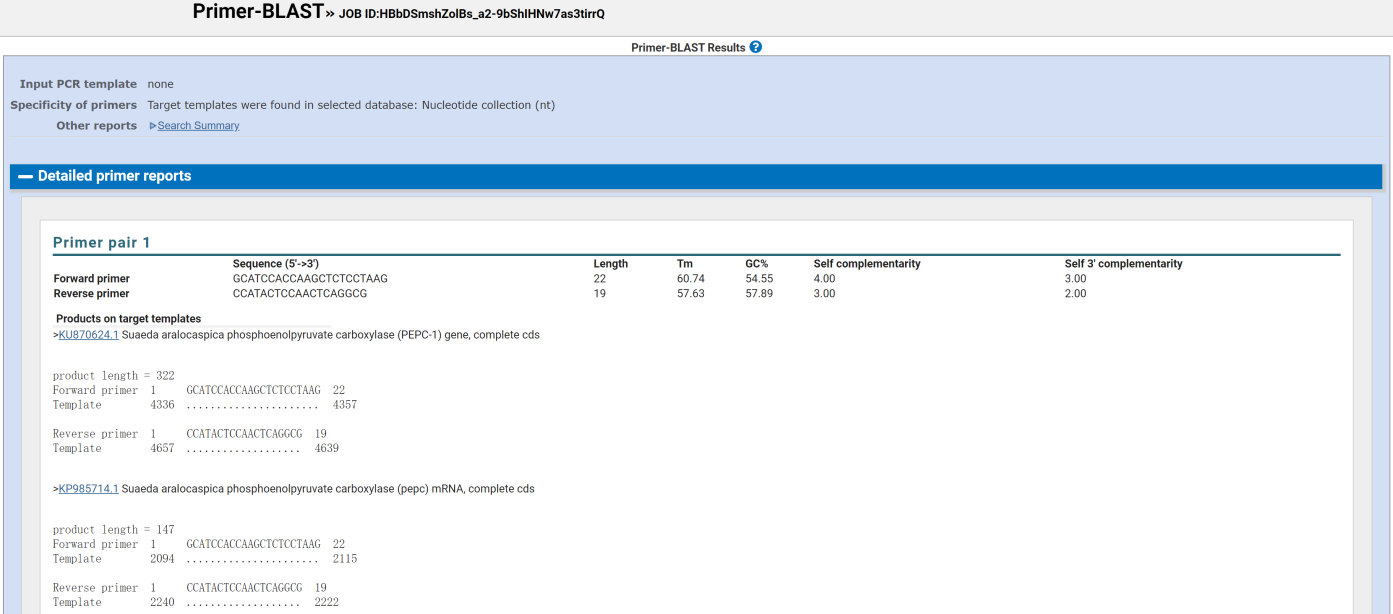

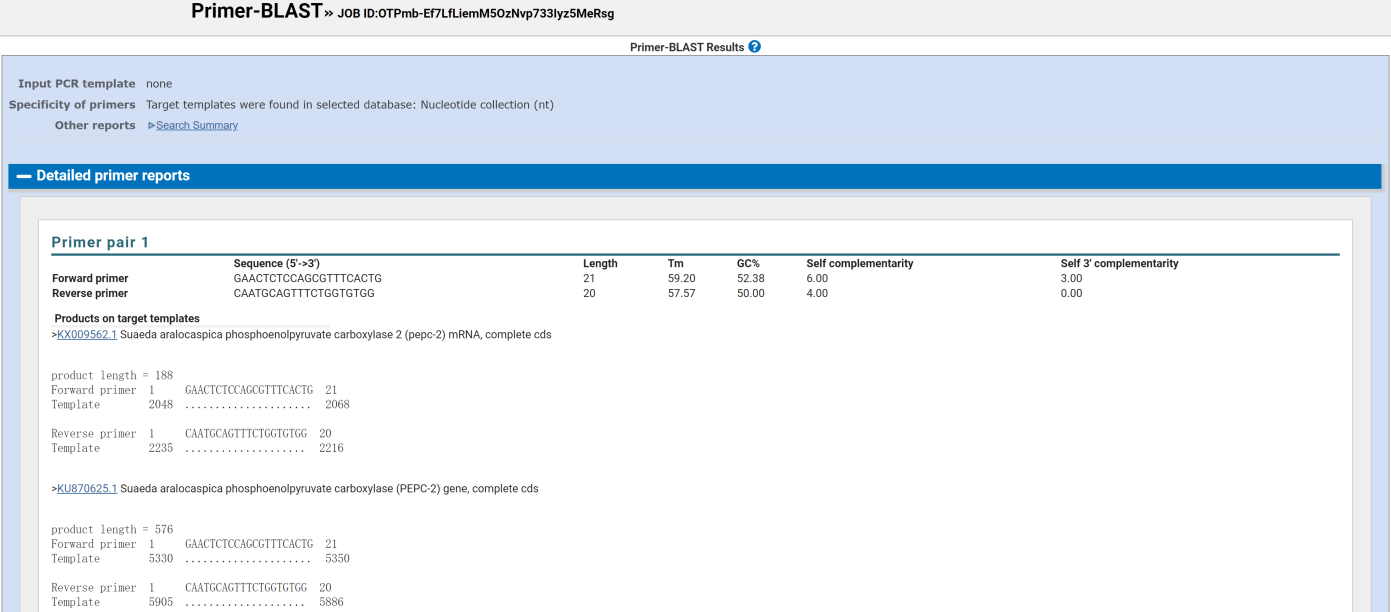


*SaPEPC-1*

*SaPEPC-2*

(ii) PCR amplification results of *SaPEPC-1* and *SaPEPC-2*


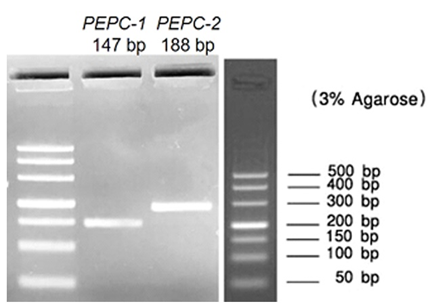


(iii) Melt curve of *SaPEPC-1* and *SaPEPC-2*


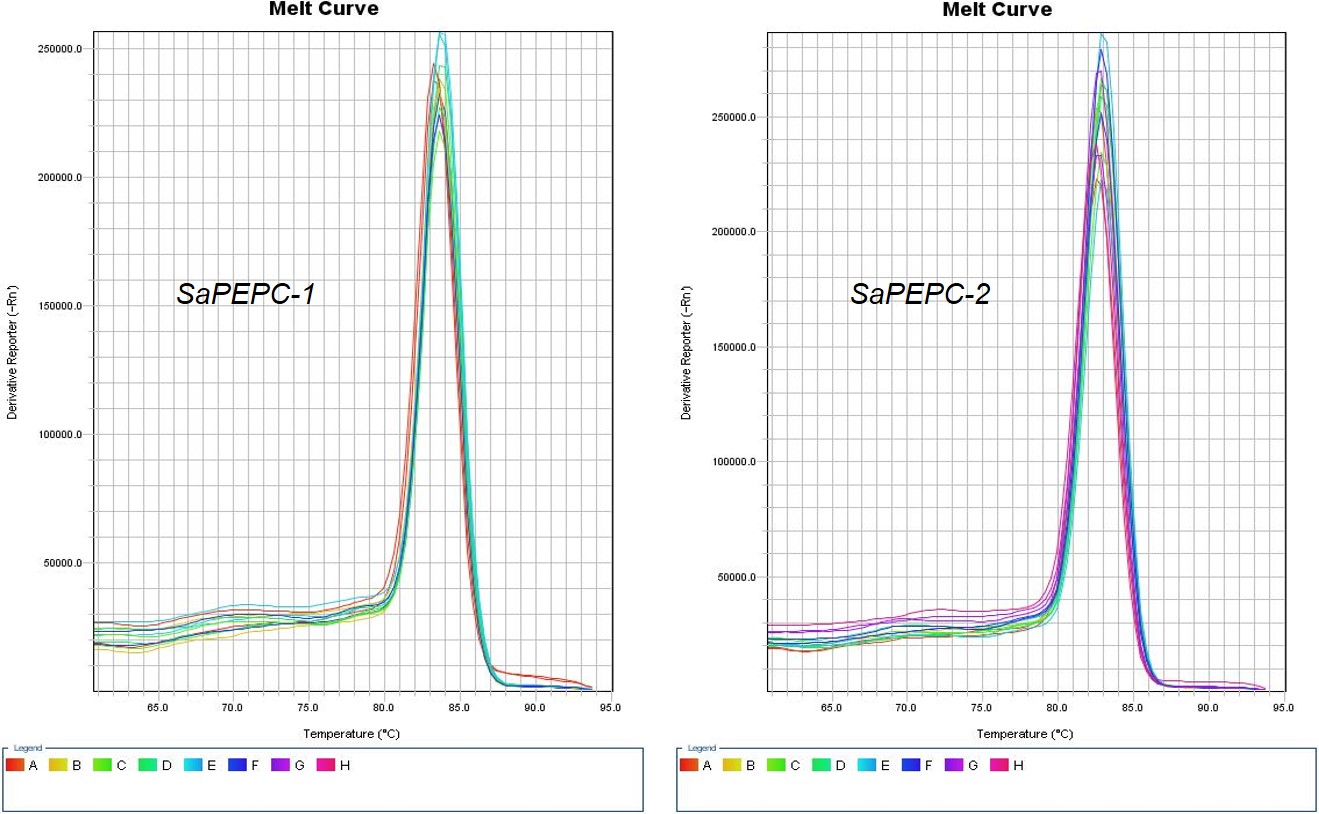


1. **qRT-PCR Primer efficiency (standard curve)**

The standard curves of *SaPEPC-1* and *SaPEPC-2* are showed below. The correction coefficient (R^2^) and amplification efficiency (Eff%) of the standard curve are shown in red font.


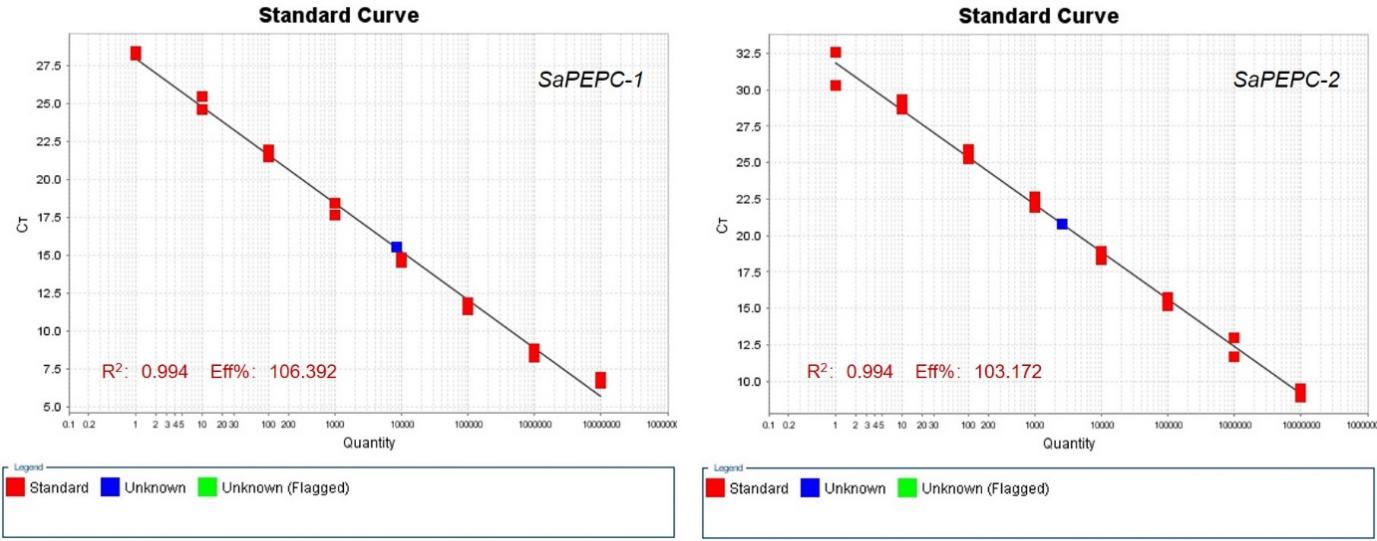


1. **RNA integrity values**

In the present study, the total RNA integrity of all samples in Figure 7-9 were detected by agarose gel electrophoresis, and the concentration and absorbance ratio (A_260_/A_280_ and A_260_/A_230_) of RNA were also determined. The results of total RNA identification under salt stress (0, 100, 300, 500 mmol ·L^-1^ NaCl) in light and dark conditions are shown below.


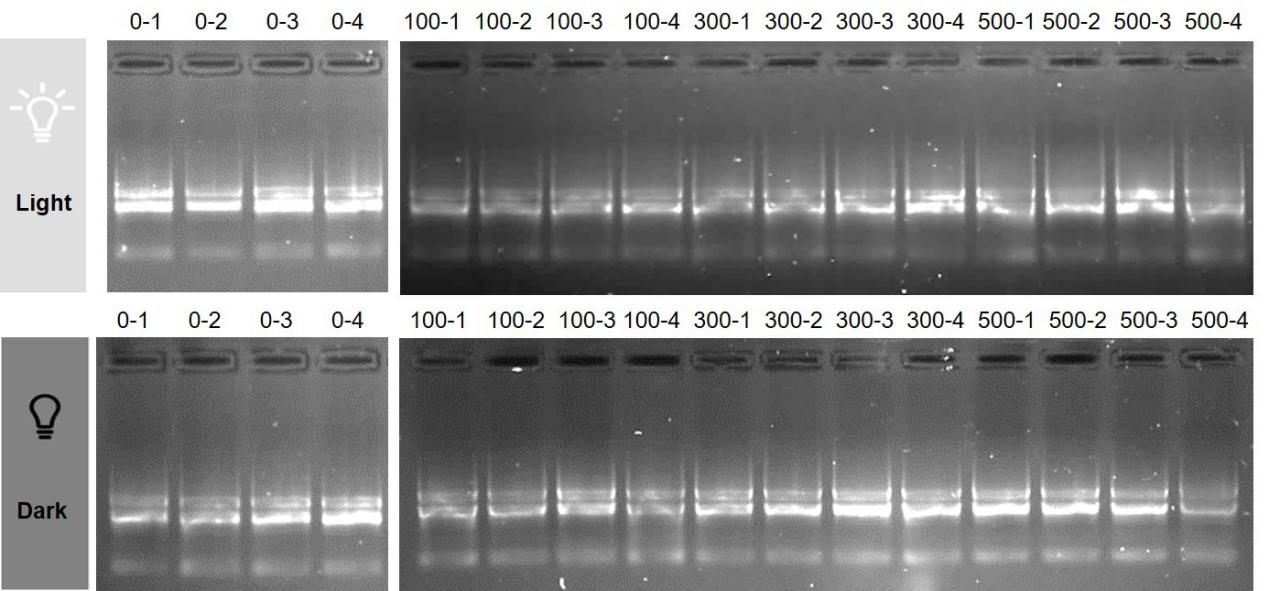


Table. RNA concentration and absorbance ratio of the seedlings under salt stress in light and dark conditions

| Light  sample | A_260_/A_280_ | A_260_/A_230_ | Concentration (ng/μL) | Dark  sample | A_260_/A_280_ | A_260_/A_230_ | Concentration (ng/μL) |
| --- | --- | --- | --- | --- | --- | --- | --- |
| 0-1 | 2.21 | 2.25 | 113.5 | 0-1 | 2.17 | 2.26 | 437.2 |
| 0-2 | 2.17 | 2.23 | 417.5 | 0-2 | 2.18 | 2.25 | 419.8 |
| 0-3 | 2.17 | 2.13 | 512.5 | 0-3 | 2.20 | 2.25 | 263.8 |
| 0-4 | 2.23 | 2.27 | 846 | 0-4 | 2.17 | 2.29 | 403.1 |
| 100-1 | 2.29 | 2.21 | 444.5 | 100-1 | 2.20 | 2.22 | 309 |
| 100-2 | 2.25 | 2.19 | 475 | 100-2 | 2.25 | 2.17 | 279.4 |
| 100-3 | 2.33 | 2.26 | 346 | 100-3 | 2.21 | 2.18 | 284.7 |
| 100-4 | 2.30 | 2.22 | 477.5 | 100-4 | 2.20 | 2.28 | 402.3 |
| 300-1 | 2.23 | 2.24 | 423 | 300-1 | 2.23 | 2.27 | 377.3 |
| 300-2 | 2.24 | 2.17 | 514 | 300-2 | 2.19 | 2.29 | 363.1 |
| 300-3 | 2.22 | 2.21 | 449 | 300-3 | 2.20 | 2.22 | 348 |
| 300-4 | 2.46 | 2.16 | 366 | 300-4 | 2.16 | 2.31 | 313.5 |
| 500-1 | 2.28 | 2.21 | 805.5 | 500-1 | 2.21 | 2.19 | 369 |
| 500-2 | 2.42 | 2.24 | 741.5 | 500-2 | 2.19 | 2.23 | 332.4 |
| 500-3 | 2.26 | 2.18 | 685 | 500-3 | 2.22 | 2.19 | 303.5 |
| 500-4 | 2.24 | 2.27 | 552 | 500-4 | 2.20 | 2.25 | 292.5 |

1. **no-RT control, no-template control values**

In the present study, the cycle threshold (Cq) value of no-RT and no-template control for the *SaPEPC-1* gene was 30.71 and 34.17, respectively, while that of *SaPEPC-2* were both “Undetermined”.

1. **Multiple reference genes with stability values (M)**

We systematically evaluated the expression stability of six traditionally used reference genes (e.g. *β-actin*, *β-TUB*, *GAPDH*, *UBQ*, *18S rRNA* and *28S rRNA*) in *S. aralocaspica* under different experimental conditions by three analytical programs (e.g. geNorm, NormFinder and BestKeeper), and assessed as well as ranked the stability of the reference genes by expression patterns. The relevant results have been published in PeerJ by Cao et al., (2016) (DOI 10.7717/peerj.1697), which revealed that *β-TUB* served as the most stable reference gene for all the sample pools, so we chose *β-TUB* as the reference gene in the present study. The stability values (M) of six reference genes based on geNorm algorithm was showed below.


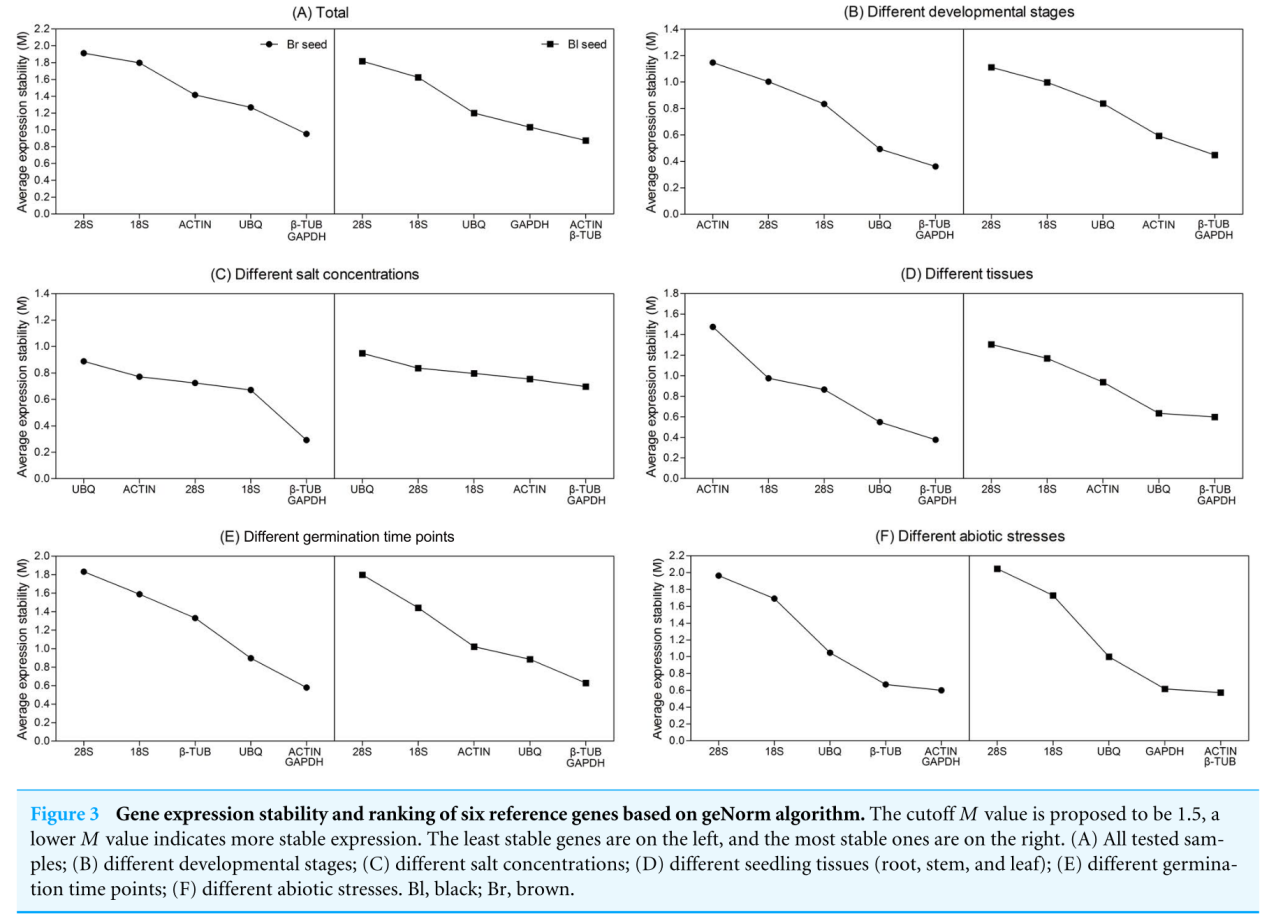

Supplement: Supplementary file 10 [file Data_Sheet_1.docx]
